# BAC-End Microsatellites from Intra and Inter-Genic Regions of the Common Bean Genome and Their Correlation with Cytogenetic Features

**DOI:** 10.1371/journal.pone.0101873

**Published:** 2014-09-25

**Authors:** Matthew Wohlgemuth Blair, Juana Marcela Córdoba, Claritza Muñóz, Deissy K. Yuyó

**Affiliations:** 1 Departamento de Agronomía y Ciencias Agricolas, Universidad Nacional de Colombia, Km 12 via Chapinero, Palmira, Colombia; 2 Department of Agriculture and Natural Sciences, Tennessee State University, Nashville, Tennessee, United States of America; 3 CORPOICA, Km 14 via Mosquera, Mosquera-Cundinamarca, Colombia; 4 Generation Challenge Program, Tropical Legumes I, c/o CIAT, Cali, Colombia; 5 Departamento de Agronomía Universidad Nacional de Colombia, Facultad de Agronomía, Bogotá, Colombia; CIRAD, France

## Abstract

Highly polymorphic markers such as simple sequence repeats (SSRs) or microsatellites are very useful for genetic mapping. In this study novel SSRs were identified in BAC-end sequences (BES) from non-contigged, non-overlapping bacterial artificial clones (BACs) in common bean (*Phaseolus vulgaris* L.). These so called “singleton” BACs were from the G19833 Andean gene pool physical map and the new BES-SSR markers were used for the saturation of the inter-gene pool, DOR364×G19833 genetic map. A total of 899 SSR loci were found among the singleton BES, but only 346 loci corresponded to the single di- or tri-nucleotide motifs that were likely to be polymorphic (ATT or AG motifs, principally) and useful for primer design and individual marker mapping. When these novel SSR markers were evaluated in the DOR364×G19833 population parents, 136 markers revealed polymorphism and 106 were mapped. Genetic mapping resulted in a map length of 2291 cM with an average distance between markers of 5.2 cM. The new genetic map was compared to the most recent cytogenetic analysis of common bean chromosomes. We found that the new singleton BES-SSR were helpful in filling peri-centromeric spaces on the cytogenetic map. Short genetic distances between some new singleton-derived BES-SSR markers was common showing suppressed recombination in these regions compared to other parts of the genome. The correlation of singleton-derived SSR marker distribution with other cytogenetic features of the bean genome is discussed.

## Introduction

Grain legumes are part of a large plant family with special characteristic of symbiotic nitrogen fixation and high protein seeds [1]. As a result, they are important for the human diet and for the nitrogen cycle of various agricultural systems. *Phaseolus vulgaris* L., common bean, is the grain legume with highest consumption around the world and plays an essential role in food security in the developing countries of Central America and Eastern or Southern Africa [2].

Common beans have many diverse landraces, modern cultivars and wild relatives within and outside the species limits of *Phaseolus vulgaris* [3]. Wild common beans, of the same species and therefore in the primary genepool, are found from Northern Mexico to Northern Argentina [3]; however, the crop was domesticated in two main regions: Mesoamerica and the southern Andes mountains [4]. Cultivars of common bean are especially diverse as many commercial classes exist for different grain types [2].

The use of molecular markers in common bean began in the 90’s with the first genetic maps using RFLPs (restriction fragment length polymorphism) [5], [6]. Since then, molecular markers have been widely used in linkage map development [7]–[9], or mapping together with genome characterization [10]–[12] or QTL identification [13], [14]. The most commonly used markers have been simple sequence repeats (SSRs) and now single nucleotide polymorphism (SNPs) that have been developed recently [15].

The first genetic map with SSR markers was published by Yu et al. [16] using an F_7_ recombinant inbred line (RIL) population for the cross between BAT93 and JaloEEP558. A saturated genetic map based on SSR markers was made by Blair et al. [17] for another inter-gene pool RIL population in the F_11_ generation derived from the cross of DOR364×G19833 using gene-derived and genomic microsatellites. At present, the BAT93×JaloEEP558 and DOR364×G19833 maps are the most saturated maps for various marker types, including gene-derived and genomic SSRs [8]–[12]. The microsatellite markers included in the second map corresponded to markers identified from databases [16]–[18], SSR-enriched libraries [7], [19], [20], non-enriched small insert libraries [10], EST sequences [21] and BAC-end sequences (BES) [11], [12].

The number of SSRs developed lately has made it possible to have a linkage map for DOR364×G19833 based exclusively on microsatellites [7], [10], [12]. However, the inclusion of EST, rDNA, RFLP and SNP markers [9], [11], [17], [22], [23], has allowed further comparisons between genetic, physical and cytogenetic maps. Indeed, comparison of genetic and cytogenetic maps for common bean started with the research of Pedrosa et al. [24] who used direct hybridization of pooled RFLP clones to position these markers relative to 45S and 5S loci on the mitotic chromosomes of cultivars Calima and Saxa using differentially-labeled fluorescent probes for *in situ* hybridization (FISH). The pool positions were identical in both cultivars except for the number, size and position of the 45S rDNA clusters. No correlation was found between chromosome and linkage group sizes, however the clustering of clones on the genetic maps was correlated with their physical proximity.

The main disadvantage of the first study (a lack of clones and low specificity) was overcome by Pedrosa-Harand et al. [25] with the use of large insert genomic clones such as bacterial artificial chromosomes (BACs) in addition to other single-copy, genomic RFLPs for FISH evaluation. The results with markers from linkage groups B03c, B04b and B07a in the genotype BAT93, a Mesoamerican gene pool standard for genomic studies, suggested that a large proportion of repeats were interspersed with single copy sequences in peri-centromeric, interstitial and sub-telomeric regions. The other eight linkage groups from BAT93 were cytogenetically mapped by Fonsêca et al. [26] making it possible to confirm the separation or intermingling of genes and repetitive sequences in various parts of the common bean genome.

In the present research, we were interested in expanding on the studies of Córdoba et al. [11], [12] by creating additional SSR markers from BES information (BES-SSR) based on singleton BAC clones from the physical map of Schlueter et al. [27]. These were then used for saturating the DOR364×G19833 genetic map and for filling gaps. The new genetic map was compared with the cytogenetic maps from Pedrosa-Harand et al. [25] and from Fonsêca et al. [26] and the comparative mapping between three core populations by Blair et al. [17] to help decide which BACs might be from peri-centromeric regions and which might be from more gene-rich interstitial regions. This genetic to cytogenetic map alignment was also useful for determining the overall coverage of the genome by the physical and genetic maps.

## Materials and Methods

### Development of SSR from singleton BACs

As previously reported by Córdoba et al. [11], [12], 89,017 BES sequences from the physical mapping project described in Schlueter et al. [27] were downloaded from the NCBI database (http://www.ncbi.nlm.nih.gov/) and were analyzed with AMMD software from Martins et al. [28] and BatchPrimer3 software from You et al. [29] for the frequency of SSR motifs and loci. After SSR detection in the two software programs, primer design around the SSR motif was carried out at the Primer 3 software website (http://primer3.sourceforge.net/). The conditions for design of the amplicons were as follows: PCR product sizes of between 100 and 300 base pairs (bp); primer length from 18 bp to 22 bp and primer GC-content from 45% to 55%. The annealing temperatures (T_m_) in the PCR reactions for each selected SSR marker ranged from 57 to 50°C at the initial cycle with 8 cycles of 1°C stepdown per cycle as indicated in Supplementary Table 1 (Table S1). The sets of SSR loci from the AMMD and BatchPrimer3 software analyses were cross-matched to the physical map of BAC clones from Schlueter et al. [27] found at the *Phaseolus* WebFPC database (http://phaseolus.genomics.purdue.edu) and only those associated with singleton BACs were used further. These new SSR markers were novel and complementary to markers from contigged BACs which were already evaluated for polymorphism and mapped in our previous BES-SSR marker studies [11], [12] and were named as part of the BMb series described in these earlier studies.

**Table 1:** Number of genic and non-genic BES-SSR markers from singleton BAC clones designed with BatchPrimer3 and AMMD that were monomorphic, polymorphic or eliminated due to amplification problems in the parental genotyping.

BES-SSRmarker set	Total numberof SSRs	EliminatedSSRs	Monomorphicmarkers	Polymorphicmarkers	Polymorphismrate (%)^a^
gene-based,BatchPrimer3-SSRs	32	4	4	24	85.7
non-gene based,BatchPrimer3-SSRs	364	82	103	179	63.4
**Subtotal**	396	86	107	203	65.5
gene-based,AMMD-SSRs	66	10	26	30	53.6
non-gene basedAMMD-SSRs	437	124	191	122	39.0
**Subtotal**	503	134	217	152	41.2
**TOTAL**	899	220	324	357	52.4

a In the calculation of the polymorphism rate only monomorphic and polymorphic markers were included.

### Parental polymorphism and genetic mapping analyses of new microsatellites

In initial screening, the new markers were evaluated on the mapping population parents involving all new markers for SSR loci with AT-rich or GA repeat motifs. PCR reactions and band visualization on polyacrylamide gels using the silver staining method and genetic mapping were carried out following the procedure of Córdoba et al. [11]. The markers that were polymorphic between the parents DOR364 and G19833 were then evaluated on all the individuals of the RIL mapping population described in Blair et al. [17]. This population was in the F_2_ derived, F_9∶11_ generation created by single-seed descent from the inter gene-pool cross between DOR364 (Mesoamerican)×G19833 (Andean).

The software program MapDisto v.1.7 [30] was used for genetic mapping. New genetic markers were placed on linkage groups with a minimum LOD of 3.0 using the “add loci” command in this software and the BES-SSR markers from Córdoba et al. [11]. Then, the markers from this study were combined with SSR markers from Córdoba et al. [12] and from Blair et al. [17] and the RFLP markers from Blair et al. [7] including 40 Bng series markers and 2 D series markers.

The best marker order for each linkage group was determined with “ripple function” and a LOD >3.0. Genetic distances were based on recombination fraction using the Kosambi function to estimate map distances from recombination values and the distances were given in centiMorgan (cM) units. All genetic mapping was confirmed with the command for “best order in MapDisto v. 1.7” [30].

### Analysis of BAC ends

The 3′ and 5′ BAC end sequences from the singleton BACs with BES-SSRs were analyzed to establish if they were homologous to expressed gene sequences or not as had been done with contigged BACs with BES-SSRs in our previous studies [11], [12]. Two approaches were used: in the first approach, the masked BES were searched against the NCBI non-redundant protein database using a BLASTX with a cutoff value equal to 1E-3. In the second approach, which was more specific for *Phaseolus vulgaris*, the aforementioned BES sequences were used in BLASTN searches against common bean expressed sequence tags (ESTs) from various studies of gene expression in the crop [31]–[48]. In this case, the BLASTN searches were performed with an E-value threshold of 1E-30. Prior to BLAST analysis, all BES were masked for transposons, simple repeats and low complexity DNA using RepeatMasker. The BES sequences and corresponding BES-SSR loci with positive hits against the ESTs or the NCBI non-redundant protein database were defined as genic.

### Integration of the genetic and cytogenetic maps

The linkage groups of the DOR364×G19833 genetic map in this study were assigned to the corresponding chromosomes by comparisons of the pooled RFLP markers from the Bng series that were located on the genetic map and were also hybridized by FISH to each chromosome [25], [26]. Some Bng loci from those studies were not mapped but were assigned to positions on the present genetic map by comparison of the RFLP probe pools and neighboring RFLPs (Bng) markers or RFLP-based SNP markers from the genetic maps of Blair et al. [17], Freyre et al. [22] and Galeano et al. [23], [49].

## Results

### Identification of SSRs in BES sequences

The computer programs used to search for SSRs in the common bean BAC-ends corresponding to singletons found different numbers of SSR loci (Table 1). BatchPrimer3 found 396 SSRs in total corresponding to the singleton BAC clones most of which had di-nucleotide based motifs (44.4%) or tri-nucleotide based motifs (30.6%) with the remainder of larger motif size. The most common motifs were the AT- rich, AT/TA (90 loci), ATA/TAT (44 loci) and AAAAT/TTTTA (20 loci) repeats. Considering microsatellite length, class II SSR loci (between 5 and 10 repeats) were slightly more common with 58.7% than class I SSR loci (longer or equal to 10 repeats) with 41.3%. AG/TC repeats were the next most common after AT/TA repeats, and were considered for marker development along with ATA/TAT tri-nucleotide motifs. Meanwhile AC/TG and CG/GC repeats were very uncommon and were not used for marker development.

The software AMMD identified a total of 503 SSRs in the BES sequences. Di-, tetra- and tri-nucleotides were the repeat types most often found, with percentages of 37.2, 35.0 and 26.8%, respectively. The remaining SSRs were either penta- or hexa-nucleotide repeats. The most common repeat types were AT-rich SSRs such as AT/TA (88 loci) or AAAT/TTTA (78 loci), but also included AG/TC (74 loci) and AGA/TCT (60 loci) repeats. Most were class II repeat length (data not shown).

The next step was to determine if the BES-SSR loci were from gene-based or non-genic sequences. This was done by taking the 1,758 sequences from the 3′ and 5′ BAC- ends of the 899 BACs with SSR loci and comparing their sequences first to all known ESTs and then to all known proteins for common bean. In the first analysis, 9.8% of sequences (173) were found to have positive hits. In the second analysis, 20% of sequences (352) were positive. When these two lists were collated for duplicates, 189 out of 1,758 independent sequences were identified as associated with gene sequences. Therefore most of the BES analyzed were found to be non-genic (89.3%). The division of gene-derived and non-genic BES-SSR loci identified with each software program is found in Table 1 and corresponds to the matches where the end of an SSR containing BAC clone also had a hit to the gene-coding sequence, where 98 BES-SSR markers were gene-derived (10.1%) and 801 were non-genic (89.9%). Out of 396 loci identified with BatchPrimer 3 software, 8.1% (32) were gene-derived. Out of 503 identified with AMMD software, 13.1% (66) were gene-derived.

When the BES-SSR were cross-matched to the physical map from Schlueter et al. [27] a total of 553 BES-SSR were from contigged BACs and 346 BES-SSR were from singleton BACs. In all but 15 cases, the singleton BACs had one SSR locus at either one end or the other of the BAC. The repeat types, repeat motifs and gene-associations of the singleton BAC-derived SSR loci were similar to those from contigged BACs (data not shown). Since the BES-SSR from contigged BACs had been prioritized in our previous analysis [11], [12], here we were interested in the subset of new markers from singleton BAC clones. We included the markers developed with both software programs described above for polymorphism screening and genetic mapping.

### BES-SSR parental polymorphism survey

With different algorithms 166 markers were designed by BatchPrimer3 and 180 by AMMD. The full total of 346 markers were added to the BMb series for Bean Microsatellites from BACs begun in the studies of Córdoba et al. [11], [12] but with the addition of letter ‘s’ standing for singleton. Therefore the new series was called BMb(s) and all the singleton BES-SSR were distinguished from contigged BES-SSR by this final letter “s” after the numbering.

The 166 SSRs from BatchPrimer3 were numbered consecutively between BMb625s to BMb875s with some intervening non-singleton BES-SSR. These markers were selected because they had AT and AG di-nucleotide motifs (106 primer pairs) or ATA, ATT and AGA tri-nucleotide motif (60 primer pairs) SSR loci. Out of the 166 microsatellites of this group, 28 were found in gene associated BES and 138 were from non-genic BES. The other group of 180 selected SSR were from the AMMD software. These markers were also denoted as BMb markers and ended with the letter “s” but were numbered consecutively between BMb1563s and BMb1742s. The 180 AMMD markers included di- and tri-nucleotide based SSR mostly with class II, AT-motif repeats. The primer pairs for the BatchPrimer3 and AMMD designed markers and respective SSR motifs are found in Table S1. In the molecular characterization of the selected 166 BatchPrimer3 SSR, a total of 108 markers presented clear and easily interpreted banding patterns of the correct or expected size range in base-pairs. Another 56 markers had ghost or stutter bands on polyacrylamide gels and were not considered useful or reliable for consistent mapping. A reduction in functional SSR markers is typical in any SSR discovery study [8]–[12].

The two groups of BES-SSR markers made from singleton BAC clones were evaluated in the parental survey. The polymorphism rate for the 108 Batchprimer3 SSR markers was 60.1% (65 markers) and this was associated with the SSR repeat type and motif class. Specifically, di-nucleotide repeat SSR with more than 10 repeats showed a higher polymorphism, with 65.6% of the di-nucleotides polymorphic while 34.4% were monomorphic. Notably, 69.8% of class I SSRs were polymorphic versus 30.2% that were monomorphic. Genotyping of the parental genotypes for the 180 AMMD derived SSR markers revealed a polymorphism rate of 43.4% with class I SSRs being more polymorphic (57.1%) than class II SSRs, representing 78 and 102 markers respectively. No relation was found between the repeat type and polymorphism in this case. A total of 52 of the AMMD derived markers were gene-derived and 128 were not from genes.

### Genetic mapping of the BMb(s) markers

Across the sets of markers developed with both software programs (AMMD and BatchPrimer3) a total of 143 SSRs were found to be polymorphic between the DOR364×G19833 parents and were amplified over the entire population. Of these, 106 had normal segregation and were placed on the genetic map for this cross. These new markers, together with 165 BES-SSR markers from Córdoba et al. [11], [12], 122 SSR markers from Blair et al. [7], [10], [17] and 42 RFLP (Bng and D) markers, produced a map of 435 single-copy marker loci with a total genetic distance of 2290.8 cM and an average distance between markers of 5.2 cM (Table 2).

**Table 2:** Information about the number (**N°**) of microsatellite markers included in the DOR364×G19833 genetic map and the physical length, genetic length and the Kbp/cM ratio of each linkage group/chromosome (LG/Chr.).

LG/Chr.	Totalnumberof markers	N° non-BES-SSRs^a^	N° BES- SSRpreviouslymapped^b^	N° singletonBES-SSRmarkers	N° Bngmarkers	Physicallength(Mbp)^c^	GeneticLength(cM)	Kbp/cMRatio
B01h/Pv01	50	9	22	15	4	58.09	230.5	252.1
B02d/Pv02	65	22	21	17	4†	57.14	288.2	198.3
B03c/Pv03	41	11	18	7	5	60.12	202.6	296.8
B04b/Pv04	36	13	12	9	2	55.88	159.0	351.6
B05e/Pv05	29	8	8	11	2	51.36	163.0	315.1
B06g/Pv06	29	7	11	4	7	46.16	170.1	271.3
B07a/Pv07	35	9	14	5	7	61.19	246.4	248.3
B08f/Pv08	47	8	23	15	1	63.62	190.2	334.5
B09k/Pv09	37	13	9	11	3†	60.39	269.4	224.1
B10i/Pv10	27	8	16	2	1	65.02	171.2	379.8
B11j/Pv11	39	14	11	10	4	58.03	200.2	289.8
**TOTAL**	**435**	**122**	**165**	**106**	**42**	**637**	**2290.8**	**278.1**

a The non-BES SSRs include the Pv markers from Yu et al. [16], BM from Gaitan et al. [19], BMd from Blair et al. [17] and BMa from Blair et al [7].

b Information from Córdoba et al. [11], [12].

c Information from Fonsêca et al. [26].

† 2 linkage groups with an additional D marker RFLPs in addition to Bng RFLPs.

Linkage groups contained between 27 (B06g) and 65 (B02d) loci (Table 2). The distribution of the new singleton BMb(s) markers was not random between the linkage groups according to a chi square test comparing even distribution on every linkage group versus uneven distribution per linkage group (χ^2^ = 24.3 p-value<0.05). This statistical test is applicable in the case of common bean where all chromosomes are approximately similar in physical length. The minimum number of new BMb(s) markers (2) was observed on B10j and the maximum number (17) on B02d. We also found that the distribution on different linkage groups for gene-derived versus non-genic BES-SSR markers was variable 1 to 14 and 13 to 28, respectively (Table 3).

**Table 3:** Number of genic and non-genic BES-SSR markers mapped in each common bean linkage group.

LG	N° gene-basedBES-SSRmarkers	%	N° non-gene basedBES-SSRmarkers	%	TotalBES-SSRmarkers
B01h	14	37.8	23	62.2	37
B02d	10	26.3	28	73.7	38
B03c	9	36.0	16	64.0	25
B04b	1	4.8	20	95.2	21
B05e	3	15.8	16	84.2	19
B06g	2	13.3	13	86.7	15
B07a	3	15.8	16	84.2	19
B08f	10	26.3	28	73.7	38
B09k	5	25.0	15	75.0	20
B10i	5	27.8	13	72.2	18
B11j	6	28.6	15	71.4	21
**TOTAL**	**68**		**203**		**271**

**Figure 1:**
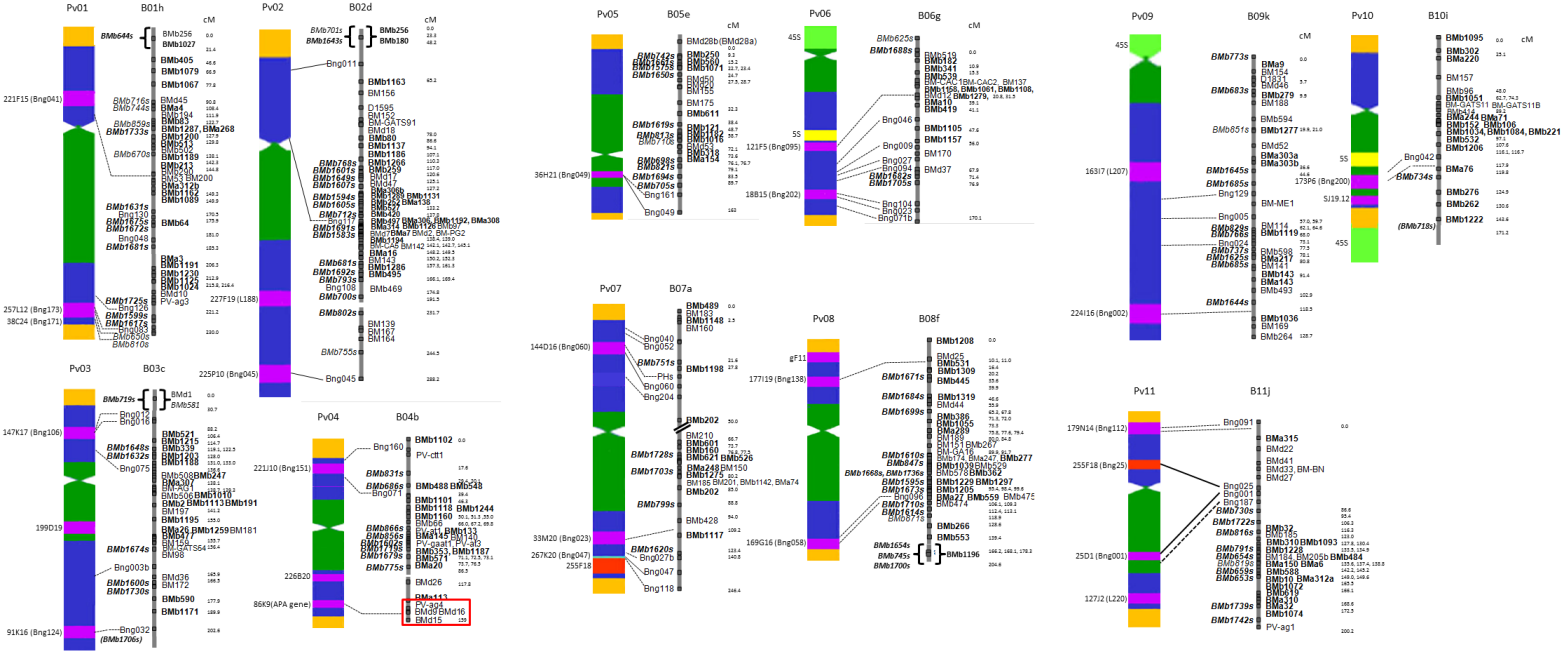
Comparison between common bean genetic and cytogenetic maps. The genetic map for DOR364×G19833 is shown on the right with previously mapped contig-based BMb markers on the right side of the bar and singleton-derived BMb(s) markers and Bng markers to the left side of the bar. Non-gene derived BMb markers are in bold while gene-based markers are in normal text. To the left of each linkage group genetic map is the cytogenetic map for the corresponding chromosome from Fonsêca et al. [26], where subtelomeric region gene-rich BACare in dark orange-red, single copy regions are in purple, peri-centromeric regions are in dark green, interstitial regions are in dark blue, 5S rDNA are in light yellow and 45S rDNA are in light green. The integration points between genetic and cytogenetic maps are indicated with continuous lines (same Bng marker) and dashed lines (linked Bng marker) based on mapping of the same or closely linked (within <10 cM) markers in Blair et al. [17]. All LGs are drawn to scale as indicated by cM distances. Red box around APA related markers on genetic map according to Blair et al. [55] align with APA containing BAC on cytogenetic map from Fonsêca et al. [26].

Inside most of the linkage groups, the new BMb(s) markers tended to form proximal rather than distal clusters observable for B01h, B02d, B04b, B05e, B08f, and B11j based on comparison of cytogenetic and genetic maps (Figure 1). Fortunately many of the Bng markers linking the cytogenetic and genetic maps were located at corresponding terminal locations on the LGs, although further linking between the two types of maps would be advisable since only 20 Bng markers were used in FISH experiments by Fonsêca et al. [26] and only 13 of these were mapped relative to the genetic map of Blair et al. [17]. The marker order for BMb or BMb(s) loci for each LG is given in Table S2.

## Discussion

Genome sequencing in common bean started with EST and BES project and has now progressed to a full shotgun sequence of the complete macromolecules representing each chromosome of G19833 (J. Schmutz, pers. communication). BAC end sequencing projects are a rich source of sequences for marker development and for linking cytogenetic, genetic and physical maps. In this case we used results from the BES project of Schlueter et al. (2008) with the 12x BAC library for the common bean Andean accession G19833 to identify 899 SSR loci based on two SSR search softwares (AMMD and BatchPrimer3). The results confirmed that multiple software analysis of sequence information can yield different numbers of class I (long repeat number) or class II (short repeat number) microsatellites as well as different amounts of compound SSRs, with a larger set of wasted primer design in BatchPrimer3 compared to AMMD. This is an aspect of marker development work that needs to be well documented and further analyzed.

By cross-matching the set of detected SSRs with the list of BAC clones that were contigged or remained as singletons in the study by Schlueter et al. (2008), we found a total of 346 independent singleton BES-SSR, 166 with BatchPrimer3 and 180 with AMMD. The present results therefore builds on the initial marker development of Córdoba et al. [11], [12] where 176 high priority di- and tri-nucleotide BES-SSR loci from contigged BACs were identified with BatchPrimer3. Likewise, the present marker set complements the 323 BES-SSR made for contigged BACs with AMMD software in Córdoba et al. [12] which were the basis for integration of physical and genetic maps of common bean. The combination of previously tested markers (499) plus those 346 presented here sums to 845 BES-SSR markers made to date for common bean and tested for polymorphism in the DOR364×G19833 mapping population parents. Polymorphism for the different groups of markers varied ranging from 65% in the study of Córdoba et al. [11], to 37.9% in this study to 37.6% in Córdoba et al. [12]. Polymorphism also varied with the program (AMMD or Batchprimer3) used to design the markers as in the study by Córdoba et al. [12] and whether the sequence was predicted to be gene-derived or non-genic as found in Table 1.

Genetic mapping of the polymorphic markers of this study and those of Córdoba et al. [11], [12] has yielded a total of 386 links between the genetic and physical maps of common bean (280 for contigged BACs and 106 for singleton BACs). Based on this genetic map we have been able to compare the distribution of BES-SSR marker types, distinguishing the mapping trends for contigged versus singleton BACs both in terms of genetic and cytogenetic distribution in the genome. The genetic map used for the entire work so far has been based on the DOR364×G19833 cross which is a core mapping population based on the selection of G19833 for the whole genome sequencing project, the large number of markers in this population and the integration with other genetic maps for the crop [17], [49].

The new integrated map presented here included not only the combination of contigged and singleton BES-SSRs from this study and from the previous two studies but also RFLP (Bng and D) markers from Blair et al. [17]. These latter single-copy markers provided a better framework for the map, making it useful for comparison with other common bean genetic maps and for linking to the cytogenetic maps of Pedrosa et al. [24], Pedrosa-Harand et al. [25] and Fonsêca et al. [26]. One advantage is that a total of 48 Bng markers plus the *Phs* locus allowed orientation of the linkage groups relative to the cytogenetic map.

In this case, we were interested in comparing the cytogenetic map with the integrated physical/genetic map for DOR364×G19833 as described here and in Córdoba et al. [11], [12]. Comparisons of cytogenetic, genetic, and physical maps and their respective markers are useful for determining which regions of each chromosome are euchromatin or gene-rich and heterochromatin or gene-poor. In this sense the Bng RFLP clones mapped mainly to the ends of both linkage groups and chromosomes which might be expected given their origin as *Pst*I-derived fragments from non-methylated and assumedly gene-rich regions of the genome.

The most important results, therefore, were found when comparing the genetic location of singleton versus contigged BES-SSR on the genetic map and the markers which have been probed onto the cytogenetic map (Figure 1). From this comparison, we hypothesize that many of the new markers from singleton BACs or BMb(s) markers were found in peri-centromeric regions on the cytogenetic map of Fonsêca et al. [26]. This was seen for the metacentric chromosomes Pv01, Pv02, Pv04, Pv05, Pv07, Pv08 and Pv11. Similarly, on the two acrocentric chromosomes, Pv06 and Pv09, the position of BMb(s) markers was mainly interstitial even if fewer markers mapped to these chromosomes. Both of these chromosomes are known to have 45S rDNA loci on their short arms which means they are not likely to have singleton BAC derived BMb(s) markers. The non BMb(s), contig-derived BES-SSR were distributed across all regions of the genome being found in non-centromeric regions of the genome and along most chromosome arms. The map order of each mapped SSR marker is provided in Table S2 and aligns with previously mapped markers and integrated maps of Cordoba et al [11], [12] and Galeano et al. [23], [49].

It was also notable that some BMb(s) markers were also found in interstitial regions of chromosomes Pv01, Pv02, Pv06 and Pv09. Almost no BMb(s) markers were found in locations corresponding to the known 5S and 45S ribosomal loci for BAT93 on the short arms of Pv06 and Pv09 or the long arm of Pv10 from Fonsêca et al. [26]. As a result, the singleton derived BMb(s) markers were generally complementary to the contig-derived BMb markers which had been one of the goals of the study: namely, to saturate the genetic and physical map and provide more linkages between these and with a cytogenetic map.

Cytogenetic versus genetic map comparisons were also valuable for comparing skewing in genetic to physical distance ratios in different parts of the genome. For example, the physically longest chromosome according to Fonsêca et al [26] was B10i with 65.02 Mb followed by B08f with 63.62 Mb and the smallest was B06g with 46.16 Mb. However, according to a chi square test (χ^2^ = 5.0 p-value>0.05) the sizes of the chromosomes were similar and differences not statistically significant. Comparison between the physical and genetic length revealed lack of concordance between the genetic and cytogenetic (physical) map length. Other observations on the distribution of the BMb(s) markers were that those with genic association tended to be in the sub-telomeric or interstitial regions while those that were non-genic tended to be in peri-centromeric regions. Therefore, within each linkage group the comparative location of BES-SSR and Bng markers may be due to non-random distribution of euchromatin (gene-rich) and heterochromatin (gene-poor) regions. Since most of the BES analyzed were non-genic this explains the biased distribution of the BMb markers especially for those from singleton BAC compared to markers from contigged BACs analyzed in the maps of Córdoba et al. [11], [12].

Apart from the physical mapping results, we discovered the proportion of the BES-SSR markers developed so far that are likely to be gene-derived based on homology with common bean ESTs or plant proteins. In this study, an unexpected high polymorphism was seen for genic BES-SSRs. Previous studies using gene-derived and non-genic markers [17] have reported that gene-based microsatellites are less polymorphic that non-genic microsatellites; however, Hanai et al [50] found the opposite. This may be related to the software used to search for SSRs as the polymorphism rates detected in the present study.

One large contribution of this research was to fill in the gaps of the previous integrated physical/genetic map of Córdoba et al. [11], [12]. It was significant that singleton-derived BES-SSRs broadened the understanding of the common bean genome through the enrichment of the peri-centromeric regions. A significant achievement of this research was further improvement of the SSR based maps for common bean based on the work of Blair et al. [7], [17] for non-BES markers. The present study also improves on the common bean integrated genetic and physical map from Córdoba et al. [11] which only included BES-SSR markers derived from physical contigs ignoring the singletons as they represented just one BAC clone instead of several overlapping BAC clones. Here we complemented that analysis, finding that singletons helped to fill out peri-centromeric regions and the inter- and intra-genic spaces of the common bean. The importance of an integrated physical/genetic map is related to its ability to cross-link molecular markers and a set of large-insert clones with a high degree of precision and accuracy. In that sense, our present study with BES-SSR derived only from singleton BAC had a focus less on physical map integration than on knowing what was missing from the physical map and why.

In practical terms, the study also increased the number of SSR markers available for common bean. With the new set of 431 singleton-derived SSR markers presented here along with the 553 contig-derived BES-SSR markers from Córdoba et al. [11], [12] the number of microsatellites for common bean now surpasses 2500 [7]–[10], [16]–[19], [50], [51]. The value of tested microsatellites is derived from their high polymorphism and the fact that they have been really proven as functional markers given that untested, *in silico* derived microsatellites tend not to amplify well.

Therefore, the empirical testing of large numbers of SSR markers creates a valuable commodity of validated microsatellites that can be used in diversity and linkage or association mapping experiments. While SNP markers tend to be easier to develop and are very abundant and amenable to high- and ultra-high throughput automation, they also are of lower polymorphism than SSR markers [52].

Finally, both genomic SSRs and singleton or contig derived BES-SSR also provide future linkages for genomic sequence to genetic map comparisons, for inter genetic map comparisons and for proper sequence assembly. This will be especially important for the further dissection of important loci like the APA locus [53] and also for the comparison of Andean (G19833) and Mesoamerican (BAT93) karyotypes as was carried out by Altrock et al. [54]. Finally the inter-genepool comparisons will be made even more meaningful by comparing reference sequences and derived genetic or physical maps.

## Supporting Information

Table S1 Primer pairs for newly-designed BMb markers for non-contigged, non-overlapping singleton BACs from Schlueter et al. (2008) and respective SSR motifs and size of amplification product.
(PDF)

Table S2 Marker order per linkage group for all BMb markers both from contigged and singleton BACs.
(PDF)
