# Supplementary material for: BAC-End Microsatellites from Intra and Inter-Genic Regions of the Common Bean Genome and Their Correlation with Cytogenetic Features
Source: PLoS One. 2014 Sep 25;9(9):e101873. doi: 10.1371/journal.pone.0101873 (PMC4177843; doi:10.1371/journal.pone.0101873)
Supplement: Table S1 — Primer pairs for newly-designed BMb markers for non-contigged, non-overlapping singleton BACs from Schlueter et al. (2008) and respective SSR motifs and size of amplification product. (PDF) [file pone.0101873.s001.pdf]

| Singleton, BMb marker | SequenceID (BAC Clone ID) | Left Sequence | Right Sequence | Expected Product Size | Motif and repeat lenght | PCR program |
|---|---|---|---|---|---|---|
| BMb625s | PV_GBa0003H21.f | CCACCAGAGATAGACAGAGC | CAGCACCGTTACACAAATTA | 280 | (TC)10 | 55-47 |
| BMb626s | PV_GBa0003O01.r | AATTATTATGTTGATGTTCGAT | TTCGGAGAAGAGAGAACAAA | 296 | (AT)13 | 50-42 |
| BMb628s | PV_GBa0007E16.r | TATTTGCAAAGCGAATATGA | ATCATTCACAAATTCTCCGT | 228 | (ATA)6 | 52-44 |
| BMb629s | PV_GBa0008N04.r | AAGTATCCTCAGATGGCAAA | CAAATATGGCATGTCAGTTG | 241 | (AT)17 | 54-46 |
| BMb631s | PV_GBa0009C05.r | TTCCCTTGAGAGGTTAGTGA | TCTGCGTGAAGTACTCAGTG | 269 | (CTT)7 | 57-49 |
| BMb632s | PV_GBa0009N16.r | TGATTGTTTACACATAGAGACAC | AGGCATTAAATGGGTGTATG | 269 | (TA)16 | 54-46 |
| BMb635s | PV_GBa0012I03.r | ACAAACAAGACCAGGCTTTA | AGAGAACTTATGGTCACCCA | 277 | (TA)12 | 56-48 |
| BMb638s | PV_GBa0014B24.f | CATATTGAATTTACCACGCA | ATGAAGTTTGTTGCTTCGAT | 216 | (AT)22 | 53-45 |
| BMb639s | PV_GBa0014C19.f | ATCAATCCCATTTCAAGAAC | TTCATGCAGGGAATAATTAAA | 155 | (AT)18 | 52-44 |
| BMb640s | PV_GBa0014E02.r | TGAATGTGATCTAATTGGCA | CGTTAAGGGCTTATCACATC | 254 | (TTA)8 | 53-45 |
| BMb641s | PV_GBa0014H08.f | CAAGTTTGACACGACTGCTA | TTCGGAAATTAATCATTCCT | 267 | (TAA)7 | 51-43 |
| BMb642s | PV_GBa0014J06.f | TAACACAAATGGCTTCATCA | TTTGAAGGAATATTTCAATTAAG | 288 | (AT)15 | 49-41 |
| BMb643s | PV_GBa0014J10.f | AAACAGGAATTTAACCAAAGTT | TCACACAACTACTGCGAAAC | 276 | (TAT)5 | 53-45 |
| BMb644s | PV_GBa0014O01.r | AATTCAATTAGCACGTGGAT | GAATGGGAAACCACTATTGA | 105 | (AT)12 | 54-46 |
| BMb645s | PV_GBa0014O18.r | AGAATTCAATGCGTAAGTTT | AGAGAATTTGAGGATGTTCG | 114 | (TA)14 | 52-44 |
| BMb646s | PV_GBa0015M19.r | ACATCCACATAAGCTCCAAT | GGGATGAATGTTCTCTTCAA | 283 | (TTA)4 | 54-46 |
| BMb648s | PV_GBa0017E15.r | CAGGCAGCCAGTGAGGAG | ATTTCCTAATTACCGTTGCG | 146 | (GA)32 | 55-47 |
| BMb649s | PV_GBa0017H23.f | AACCATTACACCAATCAAGTTT | TCCACGGATCTCTATTACCT | 205 | (ATA)4 | 55-47 |
| BMb650s | PV_GBa0017O21.r | GAAGGCGAAGTGAGTTAGTG | AAACGTTGTACGTCTCTCGT | 185 | (GAA)18 | 57-49 |
| BMb653s | PV_GBa0020G09.f | ATTTGTAAAGAACTCGCCAA | AGATAACAAGAACAATCATAAAG | 213 | (AT)14 | 51-43 |
| BMb654s | PV_GBa0021I21.f | CGCATCGATCAAAGATAGTC | CTCTTTCCCAACAAATGAAG | 161 | (TA)19 | 53-45 |
| BMb655s | PV_GBa0022E01.f | CCGTCTCTATCTATCAATCCA | GAGAGAGATAGGAAAGGGAGA | 207 | (TC)10 | 55-47 |
| BMb658s | PV_GBa0023L24.r | GAACGCTCTTCTTGCTTAAA | TTTACCTCCCTTCTACTTCTATG | 114 | (TA)8 | 55-47 |
| BMb659s | PV_GBa0024G16.r | AATTTGTAAAGAACTCGCCA | AAATATTTGATGTTCCATCTTT | 265 | (AT)14 | 50-42 |
| BMb660s | PV_GBa0024L19.f | GATATGGGAAGTGGTGGATA | ATGGAAATATTGTGCCTGTC | 125 | (TAA)4 | 55-47 |
| BMb661s | PV_GBa0025D19.r | AAGCAACACCCAGAGAGTTA | ACTCCATTAGACCCAATCCT | 229 | (AAG)8 | 57-49 |
| BMb663s | PV_GBa0025I02.r | AATCGAAACCAAATTCCTTC | TTCATATTGTTCAGATTATTCA | 191 | (TTA)4 | 49-41 |
| BMb666s | PV_GBa0026K03.f | TGTCCTCTTGAGCTTGATCT | CATTCCTCTCTCTTTCTCGTT | 268 | (AT)6 | 56-48 |
| BMb668s | PV_GBa0027C20.f | AACCTAGGGAAGTCCAACAT | CAAATATGGCATGTCAGTTG | 163 | (GAA)4 | 54-46 |
| BMb669s | PV_GBa0027K08.r | AAATTTCAGCAAGGTTTCAG | TCAAGTATTCTTTCACCAACAA | 106 | (TCT)7 | 53-45 |
| BMb670s | PV_GBa0028C10.r | GTGCCAGAGGAGAGAGAAG | CTCAAATTCCACCAATCAAT | 117 | (GA)11 | 52-44 |
| BMb671s | PV_GBa0028E12.f | AGATTGTTGCAGGGTAAAGA | ACACGAGTCCCAACAGATAG | 298 | (AT)26 | 56-48 |

| | | | | | | |
|---|---|---|---|---|---|---|
| BMb672s | PV_GBa0028L16.r | CGAGTGATCGAGTTTCTTTC | ATACATACCCACCAATCAGC | 268 | (TTC)8 | 55-47 |
| BMb674s | PV_GBa0029C23.r | TTAGAAGGTGGTTGCTTGAT | CCTATAGGTGTAAACTATTTGGT | 171 | (AT)21 | 54-46 |
| BMb676s | PV_GBa0030G18.r | TCAAGTCATTTCAGTGACCA | CACAATTTAACCCTAAACCCT | 261 | (TAA)6 | 55-47 |
| BMb677s | PV_GBa0030N20.f | GGAGATATCAATAATTTATGCG | CTCCTCCACTTTACTTCGTG | 287 | (TAT)5 | 51-43 |
| BMb679s | PV_GBa0031I19.f | ACTCGTGTTCTTCGCATAAT | CGTGTTATTGTTATTCAAATGG | 210 | (AT)17 | 52-44 |
| BMb681s | PV_GBa0033N02.f | GTCCCAGTAGCATGAAACAT | TTTACCTCAGAAGCAGTTGG | 135 | (AT)7 | 56-48 |
| BMb682s | PV_GBa0033P04.f | TCAATAAATTGGAACGAACA | TATTCGGTATGACTGGCATT | 226 | (TAA)5 | 52-44 |
| BMb683s | PV_GBa0035B13.f | GTTAGCTGATTTCACCCTTG | GTTAGGCTTAAAGTCCACCA | 128 | (AT)11 | 55-47 |
| BMb684s | PV_GBa0035F11.f | CGGGATAATTTAGTGATGGA | CCTGAGCTGTCACACAAAC | 229 | (TAT)14 | 53-45 |
| BMb685s | PV_GBa0035K15.r | AAAGTTTATCAAACGAGGCA | TATTTCGAATCTTTGCACCT | 240 | (TAA)16 | 54-46 |
| BMb686s | PV_GBa0035M10.f | ACTAAGAAGTCGCACACGAT | ACTATACCGCTTTGCACAAT | 247 | (AT)9 | 56-48 |
| BMb687s | PV_GBa0036A04.f | GTTTATCCGAATTTCCCTTT | GGGCAACTCATTCTAATTTC | 151 | (AT)8 | 52-44 |
| BMb688s | PV_GBa0036G05.f | TCCTCCTTTCTCTCACAGTC | AATGTGGTTGGTAAATTTGG | 265 | (AT)11 | 53-45 |
| BMb692s | PV_GBa0038A07.f | ACTAAGAAGTCGCACACGTT | GCACAATCAATAAGACCCAT | 244 | (AT)13 | 55-47 |
| BMb693s | PV_GBa0038A15.r | CATGTCTTTGGCATTGTATG | ATGGCAGTTAAAGAGGAGAA | 270 | (TAA)5 | 54-46 |
| BMb695s | PV_GBa0039B02.r | CTCCACCAAGATTGATGACT | CCTCCACCACAGTTTCTTT | 246 | (GA)6 | 56-48 |
| BMb696s | PV_GBa0039C13.f | GTTCATGGGACCTTGTAAGA | GAAATTTCACAACAATTTGACT | 289 | (AT)18 | 52-44 |
| BMb698s | PV_GBa0039K10.f | TGATAGGAAATATTAAGTTCAAA | GGTTAAAGAATAATCCCGCT | 206 | (TTA)7 | 50-42 |
| BMb699s | PV_GBa0041N17.r | CGCCAAACATTTATTAAGGA | GGGTACACGTGAAAGTAAAT | 205 | (TA)11 | 52-44 |
| BMb700s | PV_GBa0042M18.f | ATTCTTTGTGGTTGCCTATG | TGATTTACAAAGCGTAAGCC | 194 | (AT)14 | 55-47 |
| BMb701s | PV_GBa0042P10.r | CGTTACCAACGTCTTCTCTC | TATGTCTGCTCTTTCCGAGT | 176 | (GAA)17 | 57-49 |
| BMb702s | PV_GBa0043G04.f | AGGAAGCATCAACCACTAGA | CTGTGTGCCCGTTAGTTAAT | 191 | (TTA)4 | 57-49 |
| BMb703s | PV_GBa0043I04.r | TGTGCTTATTTCAGGATGTG | TAATTCCAAACACGGTTTCT | 265 | (AT)18 | 54-46 |
| BMb705s | PV_GBa0044A22.r | TTGAATGAATGCTAAAGCTG | ATTGGACAATGCTACAAACC | 172 | (TA)13 | 53-45 |
| BMb706s | PV_GBa0044E15.f | TGATGCATGATTACTTCTTGA | TTCGTGCTCAAATAATTGTC | 261 | (AAT)5 | 53-45 |
| BMb707s | PV_GBa0044L11.f | AATGGTGGTATACAGTGATGTT | TTGTTTGAGAAACGAAAGGT | 298 | (AAT)6 | 54-46 |
| BMb710s | PV_GBa0045H21.f | TTCTGAGAGAGAGGAGGACA | AAGGGTGCATAATGAAACTC | 222 | (AAG)14 | 55-47 |
| BMb712s | PV_GBa0046E12.f | CTTTCGTGACAAGGAGAAAC | AGGTTAACACGATTTGTATTTG | 177 | (AT)9 | 53-45 |
| BMb714s | PV_GBa0047C04.f | CCTGTTGAATTGTTGGAGAT | TTGTGTATTACAAAGGCACAC | 275 | (AT)30 | 54-46 |
| BMb715s | PV_GBa0048C21.f | GGTGATTATTTGGATTGTGG | TGTACGAGGTAGGAACGACT | 204 | (ATT)6 | 53-45 |
| BMb716s | PV_GBa0048E03.f | GAGGGTAAGTCCTAGAAGGG | CCAACCTTGTTGATCTCATT | 243 | (GAA)6 | 54-46 |
| BMb717s | PV_GBa0048J03.r | GTGTAACCTACGCTTCTTGG | ATGAAGCCAACTTTATGGAA | 229 | (TA)18 | 54-46 |
| BMb718s | PV_GBa0048L21.r | TTTCTTTCCTTTGTCTGAGC | CGCCAACCTTCTTTACTAGA | 135 | (AT)20 | 55-47 |
| BMb719s | PV_GBa0048P18.r | TGTTCACTTATCCTTACAAGAAA | TTTAATTAGCTACCAAGGTTT | 189 | (AAT)4 | 51-43 |
| BMb722s | PV_GBa0050A03.r | ATGCACCATAGAGGTTGTTT | ATCGTTTCGTATCGTGAATC | 153 | (TA)10 | 54-46 |

| | | | | | | |
|---|---|---|---|---|---|---|
| BMb724s | PV_GBa0050K15.r | ATAGGCACTACACCAATGCT | GAGAATGGACCTCTTGATGA | 282 | (AT)19 | 55-47 |
| BMb727s | PV_GBa0051F20.f | TGTTCAATCCCATAACCTTT | ACTTATCCACGTGTTCTGCT | 206 | (AT)23 | 53-45 |
| BMb728s | PV_GBa0051L01.r | TGTGGATTGTTATAGAATGTAGC | ATAGCTCTTGATTGATCCCA | 208 | (TA)16 | 54-46 |
| BMb730s | PV_GBa0052O04.f | GGAGAAGTTAAGGAAAGCGT | GAATATCTCACCTCAAACTAACA | 168 | (AT)14 | 54-46 |
| BMb731s | PV_GBa0053H01.r | TTACTTCTTTATTTGGGTGTT | TTATTCAACATTGTCAGCCA | 263 | (TAT)5 | 51-43 |
| BMb732s | PV_GBa0053H22.r | TAACCATATCAAGGAATCGG | AAATGTGTGAAATGGAAAGG | 271 | (TA)21 | 53-45 |
| BMb734s | PV_GBa0053P10.f | TTATGAATTAGCAAATCGCC | TTTCAAATCAACAGTTTCAAGT | 294 | (AT)13 | 53-45 |
| BMb737s | PV_GBa0054K02.f | AACAAGTCACCGATCAAGAC | GTTTGTAAATGATTCCGCAT | 225 | (AT)19 | 53-45 |
| BMb740s | PV_GBa0055D05.r | CCACTGACGTAAATTGTGTG | ATGGAGTTAGGATTACCCGT | 284 | (ATT)4 | 56-48 |
| BMb742s | PV_GBa0055H11.r | GTGATGTGATGAATTGGTGA | TGTGAACGTGAATGCTATGT | 271 | (TAT)14 | 54-46 |
| BMb743s | PV_GBa0055P11.f | ATTCAAATTTACCTTTGCGA | CCCTATTCTTATATGACGATTT | 296 | (TA)14 | 51-43 |
| BMb744s | PV_GBa0056C04.f | TATCCTTTCACAGTTCCGAC | ATGGGAGAAGAAGGAGAGAG | 196 | (TTC)6 | 56-48 |
| BMb745s | PV_GBa0056I14.f | AAAGCAATCGTAAACCATGT | ATGAGTAGCAATTTGGCAAT | 263 | (TAA)7 | 54-46 |
| BMb746s | PV_GBa0057C04.f | CTGATCAATCAACAGATTAACAT | AGTGTTGTTATCCCATTAAAGTT | 284 | (TA)26 | 53-45 |
| BMb747s | PV_GBa0057E08.r | ACTTTCAAATGTATTGCAGAA | TTAACTCAGCTCAACATTGC | 257 | (AAT)5 | 52-44 |
| BMb749s | PV_GBa0057M23.f | TCAGATTCGCCTAAATTCAT | GTACGCCCTTCCTGACCAT | 196 | (GA)7 | 53-45 |
| BMb751s | PV_GBa0058B01.r | ATGAAATCCAAATGAGATGG | AACAATCCACCAATATAGTTCC | 147 | (AT)14 | 52-44 |
| BMb755s | PV_GBa0060A20.r | CTGACTCAAACCACTCCATT | TGAATCCAGAGGAAGTGAAG | 202 | (CT)7 | 56-48 |
| BMb757s | PV_GBa0060M24.r | AAGAATGGTATTGAGTTGTTT | CATCTCTATGCTTTCGTTTACA | 216 | (TA)26 | 52-44 |
| BMb763s | PV_GBa0062J20.r | TCATATTATAAACTTGTTTAGGG | TTGGAGCTAACTGGGAATAA | 208 | (AT)11 | 50-42 |
| BMb766s | PV_GBa0064J09.f | CAAATAGGTGAAGACCTCAAA | AACAATGATAGTGGTATGGTAAA | 148 | (TA)18 | 54-46 |
| BMb768s | PV_GBa0065O21.r | GCTGTTTAAATTGTGCTTCC | GCATTGTTACCCTTAATCTTG | 266 | (TTA)12 | 53-45 |
| BMb769s | PV_GBa0066B04.r | AATCACTTGGTCCACAATTT | GTCAACAAGAGGCAAACAAT | 260 | (TA)13 | 54-46 |
| BMb771s | PV_GBa0070A04.f | AATTTGGATGTTGAATTTGG | ACCTTTCAGAAACCGTGTTA | 256 | (AT)22 | 51-43 |
| BMb772s | PV_GBa0070N02.f | ATGCCACATACAGCATAACA | TCTAACGGACACCTATCCAC | 181 | (TA)18 | 56-48 |
| BMb773s | PV_GBa0071B07.f | CCATGATCTTGATCTTATCCTT | GTTGTCCCATTGAGAGAAGA | 178 | (AT)13 | 54-46 |
| BMb774s | PV_GBa0071O10.f | GGAATGAGCCAATGGTATAA | TGATTGAAACGAAACACTTCT | 291 | (TAA)7 | 54-46 |
| BMb775s | PV_GBa0072A03.r | TCAACTGTTAAAGAAATCAGATG | GACTGCGTTAGAACGAACA | 249 | (AT)15 | 53-45 |
| BMb777s | PV_GBa0074D12.r | TTGAATAGCTTCTAGCCCTG | AACATGTTTGTCTTCCTTCC | 231 | (AT)13 | 55-47 |
| BMb779s | PV_GBa0074H15.f | ACGGATACACAAACTAGCAA | AATTTGGTTCTAAAGTTCATTG | 270 | (AT)15 | 51-43 |
| BMb780s | PV_GBa0074O04.f | GGTAGCAGAGCATATTGGAC | CGCCTAATAACACAACTTCA | 284 | (AT)21 | 54-46 |
| BMb781s | PV_GBa0076B02.r | TCCTAGCCGATTTAAGAGTG | GCTCATTCCTTGTTAATGAAA | 279 | (AAT)5 | 53-45 |
| BMb782s | PV_GBa0078L23.r | CCCTAAACCCATTCATACAA | TTAGTCACGAATCTCGACCT | 285 | (TAA)7 | 54-46 |
| BMb785s | PV_GBa0080J21.r | AATCTTACTTGAACGTCGGA | CTGCTGCTGCTAGACAATTA | 243 | (AT)17 | 55-47 |
| BMb787s | PV_GBa0083K06.f | TGTGAATGCTCCTTCTTCTT | GATGATAAAGGGTTTGTGGA | 203 | (CTT)6 | 54-46 |

| | | | | | | |
|---|---|---|---|---|---|---|
| BMb788s | PV_GBa0083M05.f | AAGGGTCTTCTTCTCCAAAC | CGGAATCCTTTATGTCTCAG | 265 | (AAG)5 | 54-46 |
| BMb791s | PV_GBa0084C22.r | ACAAGCACCAAAGAAATGAC | TTTGCACTAGTGTCATCACC | 292 | (ATA)12 | 55-47 |
| BMb792s | PV_GBa0084J05.r | CATACGAGGTCATCCAAAGT | GTACGGATACCTTGATGGAA | 236 | (TA)27 | 55-47 |
| BMb793s | PV_GBa0086J21.f | AGCAACAAGAGAGTCGATGT | ATTTGTGTTTGCTAAGGAGA | 173 | (AT)13 | 54-46 |
| BMb794s | PV_GBa0087F20.r | TTGAAGGAAATAACGGAATG | ATATCTGTTGGGCATGAAAG | 298 | (AT)11 | 52-44 |
| BMb795s | PV_GBa0087I12.r | TCTGGAAACCTCATTTCTCT | TTTCCAAATATTTCTACAACCA | 286 | (TAT)7 | 52-44 |
| BMb797s | PV_GBa0090A04.f | GGGTCATGTTCCTAGATTGA | AACTTAAGCCATCAAGTGGA | 183 | (TA)21 | 55-47 |
| BMb799s | PV_GBa0090D17.f | TACCCTTCCCTGAGAGGTAT | TGAATGTGTATTAGTGTTTGTAA | 219 | (TA)11 | 52-44 |
| BMb800s | PV_GBa0090F19.r | GAGGTTGGTGATGAAGGTAA | ACAATATCAAACGATAGGCAC | 212 | (AT)15 | 55-47 |
| BMb802s | PV_GBa0090K17.f | TAACCTAGCCTGATGAAGGA | AGTGAGAACTTTATAAGAGACCC | 222 | (AT)11 | 56-48 |
| BMb803s | PV_GBa0090L08.r | TTCTTAGAATCTCACTAAGCAAA | TCACATGCACAACTATTTCAC | 259 | (AT)11 | 54-46 |
| BMb805s | PV_GBa0091B10.f | GACAATGTTCAATCCCAACT | TTGATCAATCACTCCAAACC | 241 | (AT)28 | 54-46 |
| BMb806s | PV_GBa0091H12.r | CCCTTAAGCAGTGGTATCAA | ATTTCCAAGCCAACAAATTA | 162 | (GAA)5 | 52-44 |
| BMb807s | PV_GBa0091O20.r | GAGACACCCAGATGGAAATA | CTTATCAAGCAACCACCTTC | 160 | (AG)6 | 55-47 |
| BMb808s | PV_GBa0093G08.r | TTATCCCAATTATGCCAATC | AAATGGTCACTATATTGGTCTCT | 260 | (AAT)13 | 52-44 |
| BMb810s | PV_GBa0095I17.r | AGGCGAGTGAGTTAGTGAGA | AAACGTTGTACGTCTCTCGT | 182 | (GAA)18 | 58-50 |
| BMb811s | PV_GBa0097E09.r | CTCTCTCTCTCGGCAAAGTA | TTGGTGAATAGACTCTCGTTT | 105 | (AAT)4 | 55-47 |
| BMb812s | PV_GBa0097G02.f | TGAATAGATTGTTTAGGTTTCAA | AAGTTATTGGAATGTTGTGATTT | 251 | (TA)8 | 52-44 |
| BMb813s | PV_GBa0097J10.f | GTAGTCTTTGGCTGTTGGAG | AAATGGCTTATAGCATGGTT | 211 | (ATA)25 | 54-46 |
| BMb815s | PV_GBa0098B23.f | TGGCATGTGCTAGTTTGATA | TCGATTACAAGAAATCCACA | 233 | (AT)11 | 53-45 |
| BMb816s | PV_GBa0098F24.r | CGCATCGATCAAAGATAGTC | GTCTCTTTCCCAACAAATGA | 163 | (TA)19 | 54-46 |
| BMb818s | PV_GBa0099F05.r | CAACACTCGTCAATCGTTTA | CGTAACACAATCTGCCTACA | 295 | (AT)15 | 55-47 |
| BMb819s | PV_GBa0100D06.r | GACGTAAAGCTTCAGGTGAC | ACTCATCTGCAACAACAACA | 247 | (AG)7 | 57-49 |
| BMb820s | PV_GBa0100N12.f | GTTTAGGTAGTGGGCTTGGT | TCTTTGTACTGAGGCAGGTT | 268 | (TA)30 | 58-50 |
| BMb821s | PV_GBa0101D18.r | ACTGGATCTTGCTACCGTAA | AGCGATTTCATTTGTCTCAT | 233 | (TA)9 | 54-46 |
| BMb823s | PV_GBa0102G21.f | TAACCCTAACCCTAACCCTC | TTCTGGTGACAATGTTTCAA | 296 | (CT)20 | 54-46 |
| BMb825s | PV_GBa0103E17.f | CCCATCAATACTTGGCTATC | TGCACTGTGTGGTCTACTGT | 284 | (TAA)14 | 54-46 |
| BMb826s | PV_GBa0103G21.r | TTGAATTGGAAAGGAATGAT | GAAGTCATACCTCAACCTAAAGA | 187 | (AAT)7 | 51-43 |
| BMb828s | PV_GBa0103N18.f | GTTCACACAGAAACTGGGTT | GAAATGGTCCCAATTCAATA | 156 | (AG)7 | 52-44 |
| BMb829s | PV_GBa0104F18.f | GGTCCCATAGATAAAGACCA | CCCAATATGACACGGTAGAT | 268 | (TAT)5 | 55-47 |
| BMb831s | PV_GBa0104L06.r | AAGCCTCAAAGCACACTTAG | CAAAGGGTTCTTCACTTGAG | 253 | (AT)15 | 55-47 |
| BMb832s | PV_GBa0106H21.f | TGTAGATGAAAGCACCAATG | CTCCAACACATACGAGACG | 278 | (TAT)6 | 54-46 |
| BMb833s | PV_GBa0107E10.f | ACCGTCAAACATGATTCATAC | ATGTAGAAGTCGTGCCAAGT | 296 | (AT)20 | 55-47 |
| BMb834s | PV_GBa0107J07.r | TTGAATAGCTTCTAGCCCTG | AGGATTTGTTTCCAACAATG | 275 | (AT)13 | 53-45 |
| BMb835s | PV_GBa0107K07.r | GAGGAGAATTGGAAGGAAGT | GAGATAGAGTTTGTGCAGCC | 274 | (GA)11 | 56-48 |

| | | | | | | |
|---|---|---|---|---|---|---|
| BMb836s | PV_GBa0108B05.r | TTAAATCTTTGCCTTCTTCG | CCCAATCGGTACTGAAGTTA | 194 | (AT)9 | 52-44 |
| BMb838s | PV_GBa0109P15.f | CTTTCCTAACACCCAAACTG | AAGAGTGGAGCTGAAACATC | 211 | (AT)14 | 55-47 |
| BMb840s | PV_GBa0110A09.r | AAGCAACTTTACTCCCATTG | GAATTGTGTCGATCAAACCT | 192 | (TTA)7 | 55-47 |
| BMb841s | PV_GBa0110B02.r | CACCAAACACATTGAAGTTG | AAATGGCTATTCCAGACAGA | 151 | (TA)15 | 54-46 |
| BMb844s | PV_GBa0110G11.f | ATTGTTGGTATTATTGAGGG | TTCGGATCCGTATGTAAAGT | 279 | (TAT)8 | 51-43 |
| BMb845s | PV_GBa0110H01.f | TTTGGTTAGACCTTCCTTGA | CAGATAATTGTGGTGCATTG | 246 | (AT)13 | 54-46 |
| BMb846s | PV_GBa0110N05.f | ACCAGACAGCTGGAAGTAAA | TCCTTATTGGTTCCATTCAT | 202 | (AG)13 | 53-45 |
| BMb847s | PV_GBa0111C07.r | CCGAAGGTCACAATAGAAAC | CCAAGTTGACACGACTTTGT | 266 | (AT)15 | 55-47 |
| BMb848s | PV_GBa0111E06.f | CTTCTGAAACTGGAAGCAAC | AACGATAGAACCAAGCTCAG | 258 | (TCT)8 | 56-48 |
| BMb849s | PV_GBa0111F10.f | TGGTGGACAAGCTAAAGACT | TAAGTGAGCAACTGGAGGAT | 283 | (ATA)9 | 57-49 |
| BMb850s | PV_GBa0111F24.f | ATCTGCACCAACAAATAAGA | GGACGAAATTAAACGTGTTC | 245 | (AT)11 | 54-46 |
| BMb851s | PV_GBa0111J12.r | CCCAGTTTATAAGATGCGAT | TTGGGTATTCAATCTGAAGG | 160 | (GA)11 | 54-46 |
| BMb852s | PV_GBa0111L07.f | TGTCAAACTGGTTGAACAAA | CTAGCAAGTCTGCTCGATTT | 252 | (AT)9 | 54-46 |
| BMb853s | PV_GBa0111O11.r | TGTCTCACTTCAAATCCAAA | TTATATGTATCTCTCCATATCCA | 125 | (TA)13 | 52-44 |
| BMb855s | PV_GBa0112M01.r | AATAAACGGTAGCCTGAACA | GGTAATGAAATGCGAGGTTA | 246 | (TA)19 | 54-46 |
| BMb856s | PV_GBa0113G10.r | ACTAATTCCAAGCTTCACGA | TTGTGTTTATTTATATATTTCGG | 180 | (AAT)7 | 49-41 |
| BMb857s | PV_GBa0113K04.r | ATCCAAATTCCCAAATTCTT | GAACCAAGAGAGAGAGAGCA | 150 | (TC)16 | 52-44 |
| BMb859s | PV_GBa0114I24.f | CTTTCCAAACAAGGAAACAG | TTGCATGAAGCAGAGTAGAA | 248 | (TCT)8 | 54-46 |
| BMb861s | PV_GBa0114P23.f | TGGTCCCAATATTCAATTTC | ACACAACAAACATCAGTCCA | 288 | (TA)28 | 52-44 |
| BMb862s | PV_GBa0115B06.f | AACCTCTTTGCTCGTCATAA | TGAGGCAAGTGTTGTCTATG | 264 | (AT)24 | 56-48 |
| BMb864s | PV_GBa0118L08.r | TGTTCACAGTTTCATGCCTA | AGAGTACACGTGATTGAAACTT | 225 | (TA)35 | 56-48 |
| BMb865s | PV_GBa0119B13.r | AAGCCTCAAAGCACACTTAG | GATCACAAAGGGTTCTTCAC | 258 | (AT)15 | 55-47 |
| BMb866s | PV_GBa0120G15.f | GGGTTTGTAAGAAATCATGG | GGCTTCACCTACAGACTTTG | 212 | (TAT)15 | 53-45 |
| BMb867s | PV_GBa0121I22.r | TTTGGCAACAAGATACAAGA | CATAGGACAATTGGGAAGAA | 218 | (AT)14 | 54-46 |
| BMb868s | PV_GBa0121M02.f | GGAGTGAGGAGTTAGTTTGC | CAACTCCTTTGGTGTTGTTA | 282 | (TTA)4 | 55-47 |
| BMb871s | PV_GBa0123M20.f | CAGATGAGCGAGTTATCCTC | GTGGTGGTGTTCTTTCCTAA | 223 | (GA)11 | 56-48 |
| BMb872s | PV_GBa0123N11.f | TTTCTGTATGCAATGAGGTG | ACAAGTTAATTGCACGACG | 186 | (TA)11 | 54-46 |
| BMb874s | PV_GBa0124J07.f | CTGCTTCTTCATTTGCTTCT | TTGTAATTGATTGTGGCAAG | 250 | (TA)15 | 53-45 |
| BMb875s | PV_GBa0125G09.f | TGTTTGATAATGGTGATGTTTC | TTTCTACAGAAGCATGGTAAA | 233 | (AT)19 | 53-45 |
| BMb1563s | PV_GBa0106P04.r | GGAAGTCTCTAATCTCGTAAG | AGGTCATTGAGGTGTATATG | 344 | (AT)5 | 54-44 |
| BMb1564s | PV_GBa0079J05.f | GAGGCTTTTCACGTATCTA | GTTTAATACACTCCATGCTG | 202 | (AT)5 | 54-44 |
| BMb1565s | PV_GBa0079P20.r | GTACTGGTTTGAGAGGTAGTAA | CATCCTTATTAGTGTGTAGCAG | 251 | (AT)5 | 56-46 |
| BMb1566s | PV_GBa0080C03.r | GTAGTTCATCATTGGCTTAC | CTAAAACTCCCATGTATGAC | 180 | (AT)5 | 53-43 |
| BMb1567s | PV_GBa0081P24.r | GTCTTGCTGTAGATGGTTAG | CTCCAAGTCTCCAATATAGA | 243 | (AT)5 | 53-43 |
| BMb1568s | PV_GBa0060F12.r | GTAGTTCATCATTGCCTTAC | GACCATACCTCATTTGTAGA | 307 | (AT)5 | 53-43 |

| | | | | | | |
|---|---|---|---|---|---|---|
| BMb1569s | PV_GBa0061I23.r | CACATCTTCACTAATCACG | GTTGTCTCAATAGCTTCTCT | 138 | (AT)5 | 53-43 |
| BMb1570s | PV_GBa0070H06.f | GTCTATGTGAGGAGATATGAGA | ATCTCTCTCTGTTGGATCTG | 372 | (AT)5 | 55-45 |
| BMb1571s | PV_GBa0070D08.r | ATAGCTGCTGTGATGCTT | AACACCGTCTAGAGATATGAC | 134 | (AT)5 | 56-46 |
| BMb1572s | PV_GBa0074O05.r | GCACTCCTTCAGAATAATG | GTGTCTGTATAAGGGTTGAA | 363 | (AT)5 | 53-43 |
| BMb1573s | PV_GBa0073O14.r | AGAGGGAAATTGTTCTCTAC | GACAGTACGGTTATTCTAACAC | 257 | (AT)5 | 54-44 |
| BMb1574s | PV_GBa0047M20.r | CTTGACACTGAAGAATTAGG | GTCATGTGACCAGAAACTAT | 257 | (AT)5 | 53-43 |
| BMb1575s | PV_GBa0056G20.f | TACACTCTACCTCTTAATGCAC | CTGTACCACTATAATCACAGC | 384 | (AT)5 | 55-45 |
| BMb1576s | PV_GBa0058H22.f | GGTAGCTCATCTTGTGTATATG | GGGATGTCACTTATCCTTTA | 339 | (AT)5 | 54-44 |
| BMb1577s | PV_GBa0057M23.r | ACTTGTAGGAACACACTCTCT | CTGCTATATTACTCTCACCCTA | 123 | (AT)5 | 55-45 |
| BMb1578s | PV_GBa0044J16.f | GAACATATACGTGTGAGGAT | CCACTAATACGGCTAATACA | 358 | (AT)5 | 54-44 |
| BMb1579s | PV_GBa0043F19.f | GATGTAGTTCTTGGTGTAAGAG | CCAAACTATCTTCTGCAAC | 366 | (AT)5 | 54-44 |
| BMb1580s | PV_GBa0043J22.r | GTAGATCAAAGAGTACAGATCG | TTACCATGAAGTGGTAGATG | 237 | (AT)5 | 54-44 |
| BMb1581s | PV_GBa0033H22.f | CTGCTATAGACACTCATCTATC | GAGGGTCAAAGAGTACTGA | 236 | (AT)5 | 54-44 |
| BMb1582s | PV_GBa0033H22.f | GTACTCTTTGACCCTCATC | CTAGAGTAGTGATAGACATTGG | 341 | (AT)5 | 54-44 |
| BMb1583s | PV_GBa0026K04.r | CAACCACAAGAAGGTATTC | GTCTACCCTACAAGGATCTAA | 389 | (AT)5 | 53-43 |
| BMb1584s | PV_GBa0035L09.r | TATTGCTGATTGCTCAAGG | AGTATGGTGGTTATCTTAGCAG | 247 | (AT)5 | 55-45 |
| BMb1585s | PV_GBa0028I09.f | TTAACCAAAGGACTACCAC | GATCAGGTAAGATGTAATGG | 393 | (AT)5 | 52-42 |
| BMb1586s | PV_GBa0023O02.r | CTCTTCCCTCTTGTTTACTT | CCCTCATTGTCTTCTTTTC | 324 | (AT)5 | 53-43 |
| BMb1587s | PV_GBa0014C13.f | TACACTCTACCTCTTAATGCAC | CACTATAATCACAGCCTTTC | 378 | (AT)5 | 53-43 |
| BMb1588s | PV_GBa0008P12.r | TATTGCTGATTGCTCAAGG | AGTATGGTGGTTATCTTAGCAG | 247 | (AT)5 | 55-45 |
| BMb1589s | PV_GBa0018B08.r | GGAAGGTATTAATGTGTGG | CTGAGATAATAACCCACAAG | 247 | (AT)5 | 53-43 |
| BMb1590s | PV_GBa0020N21.r | GCTAGTTCATTGAGTTTCTG | CTCCAAAGACAAACTAGAAG | 241 | (AT)5 | 53-43 |
| BMb1591s | PV_GBa0020D13.r | AACTCAAAAGGAGAGATGAC | CTGGAACCTGTAATTGTAAG | 256 | (AT)5 | 53-43 |
| BMb1592s | PV_GBa0005G23.f | CACACATGAGAACTTAGACTC | GATAGTTGTAGTTGTAGACGTG | 167 | (AT)5 | 55-45 |
| BMb1593s | PV_GBa0008L10.f | TGTTTCTCCCTTAGAAAGAG | CTGCTATATTACTCTCACCCTA | 151 | (AT)5 | 54-44 |
| BMb1594s | PV_GBa0063D03.f | AGAGTTTCAACAGTGACATC | AATACTTTGGCTCTGTGC | 390 | (AT)6 | 55-45 |
| BMb1595s | PV_GBa0067O16.r | CCACCTACCTAAATATGGTACT | GATAAACTCTGCACTCTCTTC | 308 | (AT)6 | 55-45 |
| BMb1596s | PV_GBa0057A19.f | GTTGATGCTACTTAGTGTTACC | TAGGTTATCTCCTTGTAGAGAC | 381 | (AT)6 | 55-45 |
| BMb1597s | PV_GBa0049B22.r | GAGGGCTTCTAGTGTAAAAT | CCTCAACATACAACTTGC | 368 | (AT)6 | 53-43 |
| BMb1598s | PV_GBa0058E04.r | GTGAGAGTGTGAAACCTTTA | GTTAGCATACCACAAGGTAG | 279 | (AT)6 | 55-45 |
| BMb1599s | PV_GBa0027K19.r | GCTCATATAGCTTGTTATGC | ACATAGATGAGAGATGAGAGAC | 384 | (AT)6 | 54-44 |
| BMb1600s | PV_GBa0018B08.r | CTTGTGGGTTATTATCTCAG | CAAACAACTCAAGGTCTAAG | 344 | (AT)6 | 53-43 |
| BMb1601s | PV_GBa0112K06.r | AACACGAGAACCCAAAAC | ACCCTAATCTCTCTCAAAGTC | 318 | (AT)7 | 55-45 |
| BMb1602s | PV_GBa0080O18.f | GCTGCTAGCTAAGTTATGTATG | GGCTGTGTATATAGCGTTAAG | 233 | (AT)7 | 56-46 |
| BMb1603s | PV_GBa0101D18.r | ATCATACTGGATCTTGCTAC | CTCTTATACTTCGACACTTCTC | 331 | (AT)8 | 54-44 |

| | | | | | | |
|---|---|---|---|---|---|---|
| BMb1604s | PV_GBa0081G22.f | ATAGTGATGCCTAACATGAG | GCTCTTAGACTTATGCTGAA | 379 | (AT)8 | 54-44 |
| BMb1605s | PV_GBa0059C01.r | CTACCACTACTTCCTAATTCC | CACCAAACTACATCCAAGT | 316 | (AT)8 | 54-44 |
| BMb1606s | PV_GBa0057L03.f | GTCTACCTTAAAGTGTGTAAGC | GACTATGGTGTTTCAATGTC | 370 | (AT)8 | 54-44 |
| BMb1607s | PV_GBa0056A16.f | GACACTTTGCAAGAGAAGA | CCTAAGAAAGAATGTCACAC | 292 | (AT)8 | 53-43 |
| BMb1608s | PV_GBa0036J19.f | GTAAAAGTGGTCTTACTCCTTC | GCATAGAATATGTCCTCAGA | 382 | (AT)8 | 54-44 |
| BMb1609s | PV_GBa0038I08.f | CTATAGAGGTTGTTTTGGTG | CAATAAAGAGTACCTGAGTACC | 328 | (AT)8 | 53-43 |
| BMb1610s | PV_GBa0067P24.f | AGGTGTACGGAGTTATTTG | CTCTTCGTTTAGGTCACAT | 371 | (AT)9 | 54-44 |
| BMb1611s | PV_GBa0054K11.r | GCTGATGATATGTCTTGTCT | GTAGTGAGACCCTTACCACT | 396 | (AT)9 | 54-44 |
| BMb1612s | PV_GBa0003H02.f | GTTAACTCCCAATAGTGATG | CTATAGAATCTTGGTCCGTA | 400 | (AT)9 | 53-43 |
| BMb1613s | PV_GBa0118B14.f | GTTTATGCTCCAATGCAC | GGGACAGAAAGTGAAAGA | 366 | (TA)10 | 54-44 |
| BMb1614s | PV_GBa0041N17.r | GGGTTTGGTAAATAAGAGTC | GGGTACACGTGAAAGTAAAT | 232 | (TA)10 | 53-43 |
| BMb1615s | PV_GBa0104G14.r | GAACGTGTCACTGATTTTAC | CTTAGTCTCAAAAGTGAAGC | 380 | (TA)11 | 54-44 |
| BMb1616s | PV_GBa0101D24.f | CAGCCTTACTCTTACTAATCC | TTTCGGTCTCTATTTCTCTC | 384 | (TA)11 | 54-44 |
| BMb1617s | PV_GBa0080J21.r | CCATTCTAAAACACCTTTCC | ATATTCCTTGAGTGTGATGG | 159 | (TA)12 | 54-44 |
| BMb1618s | PV_GBa0080P15.r | GGCTGAAAAGAATCTACTAC | GTTCAACTTGTGGATTAGAC | 257 | (TA)12 | 53-43 |
| BMb1619s | PV_GBa0005G01.r | CTACAGTCCTATTGCGTTAC | AATGGGGACTCTAAGTTATC | 150 | (TA)12 | 54-44 |
| BMb1620s | PV_GBa0108K17.r | CAGTTGTTCTTCTAGTTACCC | CTTCTCATCACCTTCAAAC | 289 | (TA)14 | 53-43 |
| BMb1621s | PV_GBa0052A10.f | TCCAACCTATCACTATTCAC | TAGTTGGTAGTATTGGGGTA | 325 | (TA)14 | 54-44 |
| BMb1622s | PV_GBa0009N16.r | GTTTACACATAGAGACACACG | GAACCTAAAGAGTAGAAGATGC | 396 | (TA)15 | 56-46 |
| BMb1623s | PV_GBa0016A15.f | CACCATTATGAACAGTCAAC | TCTATCTCTCTTGCGTTCT | 396 | (TA)15 | 54-44 |
| BMb1624s | PV_GBa0076C06.r | GGTTACTGAGCATACCAAG | CGAGGACAATTCCTTTTAC | 370 | (TA)16 | 53-43 |
| BMb1625s | PV_GBa0117P10.r | CTCTTTTCAATGCTTCTCAC | AAGCAATCTAAGGTGTGG | 383 | (TA)5 | 54-44 |
| BMb1626s | PV_GBa0118M22.r | TACGGGGAATTCTAAAAGC | GTGTCTACCAATTTCACCATAG | 344 | (TA)5 | 55-45 |
| BMb1627s | PV_GBa0111B07.f | GTTCTCCTAAACTGTTCTCC | CTAGACCAAAAGCCAAAG | 359 | (TA)5 | 53-43 |
| BMb1628s | PV_GBa0094I16.r | ATTGTGTCTGGCTGTTGT | ATAATTGTTCGGACACAGG | 367 | (TA)5 | 55-45 |
| BMb1629s | PV_GBa0102D23.f | GTAGTCCCAGACTGAAAACT | GTTCTACCTCCTTTCTCAAT | 338 | (TA)5 | 54-44 |
| BMb1630s | PV_GBa0104E10.f | GGTATGTTAACCTCTGTTTCAG | GTAGGGCTCATGTTTATTTC | 235 | (TA)5 | 54-44 |
| BMb1631s | PV_GBa0103B19.r | GGGTGTTTACATACCTAATG | AAGTAGTCTAACAGTCCTTCAG | 400 | (TA)5 | 53-43 |
| BMb1632s | PV_GBa0104H24.r | TCCTGCCTCTACTGTTTTA | GGAGATCTTCTTCCTAATGT | 203 | (TA)5 | 54-44 |
| BMb1633s | PV_GBa0091L02.r | GAATCTTGATGTAGTGGTGA | TATATCTAGGTCCTCCAAAGAC | 345 | (TA)5 | 54-44 |
| BMb1634s | PV_GBa0079J12.r | TAGGAAGAAAGTGTGTTGTC | GTTCCTTCTTTGTAATACCC | 256 | (TA)5 | 53-43 |
| BMb1635s | PV_GBa0090N05.r | GGGAATATCAAAGATAGAGG | CAACTTCCACACATTTCC | 348 | (TA)5 | 52-42 |
| BMb1636s | PV_GBa0092M04.r | GAACTTGGTAACTAGAGTGTTC | TAAGTCCAGTGGTACAGTAGAT | 272 | (TA)5 | 56-46 |
| BMb1637s | PV_GBa0089K09.f | AGTGAGTTGTACATGGAGTAAG | GGAACATGTCACTTAAAGG | 150 | (TA)5 | 53-43 |
| BMb1638s | PV_GBa0080C12.r | GGCTTTTCAATCTCAATGTC | TACACGGCTCTTAAAAGGTA | 181 | (TA)5 | 55-45 |

| | | | | | | |
|---|---|---|---|---|---|---|
| BMb1639s | PV_GBa0080J12.f | CTATGCCCATCTCTATGTAA | TTGTCACTATGTACTACTGCTG | 295 | (TA)5 | 53-43 |
| BMb1640s | PV_GBa0068B18.f | GTAGCAGCAAGAGACTCAG | GTTGTTGTAGACTGAAAGTAGG | 280 | (TA)5 | 56-46 |
| BMb1641s | PV_GBa0058O05.r | CTAAATGTGATTAGCTGCTG | AGGGACTAAAGTTAATACGG | 347 | (TA)5 | 54-44 |
| BMb1642s | PV_GBa0058J04.f | GTGATTCAACTCTCAACACT | CTCTTCCTTTACTTCTTCCT | 268 | (TA)5 | 54-44 |
| BMb1643s | PV_GBa0066L18.f | ATAACGAGGTGGTCTAGATT | GGAGTTTTCAGAATTAGGAG | 378 | (TA)5 | 53-43 |
| BMb1644s | PV_GBa0060I15.f | AGTATGGTCTTACGCAATAG | TACATGAGGGTATGTTTAGG | 198 | (TA)5 | 54-44 |
| BMb1645s | PV_GBa0061L13.r | CAAGTTCATTGACCCAAC | GTTGTATCTGACGACACAGT | 147 | (TA)5 | 54-44 |
| BMb1646s | PV_GBa0055J17.f | CACACGTCTACTAAACTATGC | CTGAATTGGATGTAGGAAAC | 290 | (TA)5 | 53-43 |
| BMb1647s | PV_GBa0057D21.f | CTCCAGATGTAGGAAATATG | GAATCTCCTCTTCTAGTTACTG | 223 | (TA)5 | 52-42 |
| BMb1648s | PV_GBa0053B04.f | GTTAATGCATCATACCTGAG | GGAGATCTTCTTCCTAATGT | 341 | (TA)5 | 53-43 |
| BMb1649s | PV_GBa0048N22.r | CGGAATACGGTTCTACTTA | TTACTAGCTGTCCACGTTAT | 306 | (TA)5 | 53-43 |
| BMb1650s | PV_GBa0047A04.r | GTTAAGAAGATCCGAGAAGT | GTTTGACTAAGGCACTCTTT | 229 | (TA)5 | 54-44 |
| BMb1651s | PV_GBa0029P21.f | CCTCCTGTTTTCTCTTTAGT | TACTTCCTCACTGTTCAGTCTA | 314 | (TA)5 | 54-44 |
| BMb1652s | PV_GBa0032C17.f | GTCAGTAAGATTAAGACACTCG | GTAGGACAAATAGATGTGTAGG | 397 | (TA)5 | 55-45 |
| BMb1653s | PV_GBa0039O07.r | ACTTAGCATGAAGTTGGTG | TAGATTTGTGTCCCTATGTC | 226 | (TA)5 | 54-44 |
| BMb1654s | PV_GBa0027B14.f | AGCTTAGACTTTTGAAGGAC | AAAGCTAGAGAAACTTCCAG | 345 | (TA)5 | 55-45 |
| BMb1655s | PV_GBa0035H03.r | GTGTTGAAATGATGCTGTC | TACCTCAAAAGAGGCTAACT | 219 | (TA)5 | 54-44 |
| BMb1656s | PV_GBa0028C02.r | CAGACCTAACACTCAAATTC | TAGTATCATGGTTGACTTGC | 258 | (TA)5 | 53-43 |
| BMb1657s | PV_GBa0039O22.r | ACTGCTGGTAAAGTATTGTG | AGAGTGCTAGATAGGGAAAT | 375 | (TA)5 | 55-45 |
| BMb1658s | PV_GBa0014O15.r | GGTACCTGGTCTTCAATTA | GGAATTGGATCTCGTACT | 239 | (TA)5 | 53-43 |
| BMb1659s | PV_GBa0020M05.r | TACGGGGAATTCTAAAAGC | GTGTCTACCAATTTCACCATAG | 342 | (TA)5 | 55-45 |
| BMb1660s | PV_GBa0018O18.r | TGAGAGCTACTAACAATGAC | CTTGCATATTGTACCAGAG | 346 | (TA)5 | 53-43 |
| BMb1661s | PV_GBa0018A21.r | GAAAGAAGAGTTGTTAGTGC | ATAGTGCAAAACCTGTCTC | 309 | (TA)5 | 54-44 |
| BMb1662s | PV_GBa0017L10.f | GGTACGATGTTAAGTATGTCC | CTTGTCTTCTCTTTGTTCAC | 219 | (TA)5 | 54-44 |
| BMb1663s | PV_GBa0017N08.f | GACATTGGTACAATTGAGG | CAGTGTGAAGGTTCTAAAAG | 314 | (TA)5 | 53-43 |
| BMb1664s | PV_GBa0007B23.r | GTTGAGTTACGTGCTTACTT | ATAGGACAGAGGTCTTCTAAAC | 370 | (TA)5 | 55-45 |
| BMb1665s | PV_GBa0007N10.f | ACTTCGCAGTGTGATGAA | ACCTCTTCATTGTCTTTGTC | 391 | (TA)5 | 55-45 |
| BMb1666s | PV_GBa0112G14.r | GATGAGTGTCTCCACTTTTA | CTCTTTCTTAATGACCACTC | 338 | (TA)6 | 53-43 |
| BMb1667s | PV_GBa0096I16.r | ATGTTATTGACCCTACACAC | GTTAGATACACAGGTCAGGA | 324 | (TA)6 | 55-45 |
| BMb1668s | PV_GBa0090B16.f | GAGTGATTCCTATCTCTGTTAG | TGCTTGTAGCTGGATAGTTA | 259 | (TA)6 | 55-45 |
| BMb1669s | PV_GBa0037D07.r | CTACAAGACATAAAGCATCC | GGTGAGAGAATTGAATGAG | 393 | (TA)6 | 53-43 |
| BMb1670s | PV_GBa0023G21.r | CTACAAATTGAGCACATCTC | CATTAGCTATGGCATGTTG | 322 | (TA)6 | 54-44 |
| BMb1671s | PV_GBa0118F08.r | CTACTTGCTGAGTACACAAAC | GAGTATATATGCCACTCTGAAC | 385 | (TA)7 | 55-45 |
| BMb1672s | PV_GBa0119L15.r | TACTTTAGGTTGGTGTGAAC | ACCTACAAAGGTAGAGTTAGTG | 221 | (TA)7 | 55-45 |
| BMb1673s | PV_GBa0090L22.r | CTGAGGAATCAGTGTTTAAG | TATATGAACATGGAGAGTGG | 285 | (TA)7 | 53-43 |

| | | | | | | |
|---|---|---|---|---|---|---|
| BMb1674s | PV_GBa0062A16.r | GTGTGTTAATGCACTGTTCT | GCTGAGACGAACTAGTATTAAG | 373 | (TA)7 | 55-45 |
| BMb1675s | PV_GBa0060L01.r | TACTTTAGGTTGGTGTGAAC | ACCTACAAAGGTAGAGTTAGTG | 222 | (TA)7 | 55-45 |
| BMb1676s | PV_GBa0028B22.f | GGACTCGACTAATTTGAACT | AGATTTAGGATGCTGGAG | 321 | (TA)7 | 53-43 |
| BMb1677s | PV_GBa0008F05.r | ATCTACGTGCACTTTCTTC | GACTTGTTATCACGTTTGG | 307 | (TA)7 | 54-44 |
| BMb1678s | PV_GBa0112M13.f | GAGCCTGAAAGACAAAGTA | CCTAGGTAGTTTCCTGTAAATC | 345 | (TA)8 | 54-44 |
| BMb1679s | PV_GBa0108A12.r | AGGTTTTGGTCTATCCTAGT | AGTACATGTCTCAATCCTCTC | 334 | (TA)8 | 55-45 |
| BMb1680s | PV_GBa0105D16.f | CTAGGTGTTCACAAGTTACTG | GCTAAACTCTAGGAAAGAGAG | 275 | (TA)8 | 55-45 |
| BMb1681s | PV_GBa0086I09.r | GAACTAGCCTCATTTCTTAC | CTCTATTACTAACTGCACCAC | 279 | (TA)8 | 53-43 |
| BMb1682s | PV_GBa0106D12.f | GATTTCTCCCAAACTAGACT | AAGAGACTTACACCAAACCT | 176 | (TA)9 | 54-44 |
| BMb1683s | PV_GBa0091G14.r | GTGTGTCTCCTTCAACTTC | GAGATTGACATCTATTCGAC | 325 | (AAT)4 | 53-43 |
| BMb1684s | PV_GBa0090N05.r | GTAAAGTGCTAGAAGTGGAA | CTTTGATATTCCCAGTCC | 305 | (AAT)4 | 52-42 |
| BMb1685s | PV_GBa0080K20.f | GTTTTGACACCACTGATCTA | GAAATGTGTTACTGGATACC | 236 | (AAT)4 | 53-43 |
| BMb1686s | PV_GBa0048F09.r | CAACTACTTCTAAAACGACACC | CCCATTCAAAAGTTACGAAG | 275 | (AAT)4 | 54-44 |
| BMb1687s | PV_GBa0052D16.f | ACGAAGAAGGAGAAGTTAGT | AGATTGTAGTCTTCGGTTTC | 392 | (AAT)4 | 55-45 |
| BMb1688s | PV_GBa0020G23.r | GATCTAGACTGTAGGGTTTGT | CAAGTCTACGCTAGAATCAT | 282 | (AAT)4 | 54-44 |
| BMb1689s | PV_GBa0110G11.f | GTATTATTGAGGGGTGGA | CTATCACATGACCACTACAAG | 378 | (AAT)5 | 53-43 |
| BMb1690s | PV_GBa0080E19.f | ATAACATCTACCTGACACGA | GTTAGAAACCATAGTCTACACG | 399 | (AAT)5 | 55-45 |
| BMb1691s | PV_GBa0053G21.f | AACAGTTTGGTAGACGAAG | GTCAGAACTTAGCTGAATAGAG | 323 | (AAT)5 | 55-45 |
| BMb1692s | PV_GBa0024D11.f | CTAAGGAAAATCACCATGAC | GGTTATCACGTTAGTCTTACAC | 250 | (AAT)6 | 53-43 |
| BMb1693s | PV_GBa0096A16.r | CCTATATTGCATCTGATTGG | CTAGTGATGAAGTTTCTCTCCT | 254 | (ATA)4 | 53-43 |
| BMb1694s | PV_GBa0088H20.f | GCAGACTCACAGACCTAAA | GTTTTCACATATCTCTGCTG | 213 | (ATA)4 | 54-44 |
| BMb1695s | PV_GBa0026O07.r | ATAGGAGAAGGACTAATGGA | GCCCTACACTTATCAATTC | 352 | (ATA)4 | 53-43 |
| BMb1696s | PV_GBa0032D07.f | CCTATATTGCATCTGATTGG | CTAGTGATGAAGTTTCTCTCCT | 254 | (ATA)4 | 53-43 |
| BMb1697s | PV_GBa0116O02.f | CCTCATTAGGTAGAAGAACTGA | GAAGGCCAACAGAAACAA | 294 | (ATA)5 | 55-45 |
| BMb1698s | PV_GBa0119M24.f | GACTACTTACTTGTGCTAGACC | GCGTTCTAGTGTCTGTGTAT | 285 | (ATA)5 | 57-47 |
| BMb1699s | PV_GBa0086K15.r | TATCAGAGAATCTGCCTAGA | GACTGTATTTTCTTGGGTCT | 393 | (ATA)5 | 54-44 |
| BMb1700s | PV_GBa0028C14.f | GTGCACAAGTATGTTTCTGT | CATATATCAGACACTGACACTC | 252 | (ATA)5 | 55-45 |
| BMb1701s | PV_GBa0118H01.f | ACGTATCTTCTTGTGTTAGC | GAAGTGCACCACATATACTT | 335 | (ATT)4 | 55-45 |
| BMb1702s | PV_GBa0086C06.r | TATCTATTAGGGGACTTTCC | CACAAGGCTAACTAAGTAGAAC | 161 | (ATT)4 | 53-43 |
| BMb1703s | PV_GBa0055L01.r | GTGTAACATCCCAAACTTAG | GTTTGAGAGAGATAGGTTCTG | 360 | (ATT)4 | 53-43 |
| BMb1704s | PV_GBa0055D05.r | CACTGACGTAAATTGTGTG | ATATGGAGTTAGGATTACCC | 285 | (ATT)4 | 53-43 |
| BMb1705s | PV_GBa0028C17.r | CTATGTCACTTAAGATCTCAGC | TTGGTATCTCTCTAGAATGG | 193 | (ATT)4 | 53-43 |
| BMb1706s | PV_GBa0014M05.r | ATATCTGCAGTGTGATTAGC | CTTCTACCCCACACAATAC | 349 | (ATT)4 | 55-45 |
| BMb1707s | PV_GBa0022D21.r | TACCTCTTCCTTCTCTTCTT | CACAAGGCTAACTAAGTAGAAC | 318 | (ATT)4 | 55-45 |
| BMb1708s | PV_GBa0022D24.f | TCTACCTCTTCCTTCTCTTC | AAGAGTATGTTGACATGGAG | 355 | (ATT)4 | 54-44 |

| | | | | | | |
|---|---|---|---|---|---|---|
| BMb1709s | PV_GBa0083D24.r | GACTTTGAAGGAACTGCT | GTCATACCTAACTCGCAATA | 398 | (ATT)5 | 54-44 |
| BMb1710s | PV_GBa0091D17.r | GACTCATCTTCTTGGATTCT | GGACTAGGAATGTGAGTAAA | 381 | (ATT)5 | 54-44 |
| BMb1711s | PV_GBa0028C17.r | AGAACCATTCTAGAGAGATACC | CTACTGAAGGTTCGCTAATA | 349 | (ATT)5 | 54-44 |
| BMb1712s | PV_GBa0004F22.r | TCTTCTTGGACTTGTGTTC | AGATAGGGAACCAATGTATC | 396 | (ATT)5 | 54-44 |
| BMb1713s | PV_GBa0103A07.f | GTGATGTTCTCTACCAGAAA | CAGCACTGATTCTTCTTG | 388 | (TAA)4 | 53-43 |
| BMb1714s | PV_GBa0080P15.f | CTTCTACTTACCACACCCTAT | GTGAGGTGAGCTTAATTTG | 182 | (TAA)4 | 54-44 |
| BMb1715s | PV_GBa0027D12.r | GGATGTTAGTGATGGATAGA | GTTGTAAGGAAGGAGGAGT | 334 | (TAA)4 | 53-43 |
| BMb1716s | PV_GBa0039G01.f | CTTCTACTTACCACACCCTAT | CTACCAAACAAGTGACAGAT | 360 | (TAA)4 | 55-45 |
| BMb1717s | PV_GBa0013O17.f | GGACTAAATAGTGTCAGCTAAG | AGCTCAATAGTTCACGTTC | 328 | (TAA)5 | 55-45 |
| BMb1718s | PV_GBa0017O22.r | CCAGTGAGTCAAAGACAATA | AGTTGTGCATAGCAAGTAAC | 230 | (TAA)6 | 54-44 |
| BMb1719s | PV_GBa0056H13.f | GTGGAGAGTAAATTGAAAGC | GCACTAAAAGTATCCTTGG | 213 | (TAA)7 | 53-43 |
| BMb1720s | PV_GBa0094I16.r | GTGTCTGGCTGTTGTTCT | ATAATTGTTCGGACACAGG | 364 | (TAT)4 | 55-45 |
| BMb1721s | PV_GBa0109A18.r | CACATAGGGTAACGATACAT | TGTCTAGCCTTTCTTACTTG | 358 | (TAT)4 | 54-44 |
| BMb1722s | PV_GBa0085F16.r | CCACCTTTTCTGGTATACAT | GGAGGATTGAGTCTTAGTAAC | 377 | (TAT)4 | 54-44 |
| BMb1723s | PV_GBa0045C22.r | ACTATAACCCATCATCACAC | GGACTGATCTAATGAAAGAG | 204 | (TAT)4 | 52-42 |
| BMb1724s | PV_GBa0055D10.r | CAGAGTAGGACAACCTTTG | GTTGTTTACTGCTTCACTTC | 110 | (TAT)4 | 54-44 |
| BMb1725s | PV_GBa0013A15.f | TACCAATATGTTCGGAGAG | CAACCTTAATCAGTGTTAGC | 376 | (TAT)4 | 53-43 |
| BMb1726s | PV_GBa0015O20.f | GAGTGTCTCTTACTATCATTGG | CCTCTCTCTACAACTTTCAGTA | 209 | (TAT)4 | 55-45 |
| BMb1727s | PV_GBa0010O08.r | TACTGATTGGATGTCTAAGG | CACAGTTATCACTTTAGAGGAC | 106 | (TAT)4 | 54-44 |
| BMb1728s | PV_GBa0087I12.r | GTACCCCAAGTACTCCTATT | CAACCACTAGGTAAAATGG | 221 | (TAT)6 | 53-43 |
| BMb1729s | PV_GBa0058G08.f | CTATGTAGAGTTTGTGAAGCAG | AGATACATGGAAAGCACAC | 351 | (TAT)6 | 55-45 |
| BMb1730s | PV_GBa0107M20.f | CCTGCACTGAGATTTAATAG | CGGTCAGTTCTGAGATAAC | 258 | (TAT)7 | 53-43 |
| BMb1731s | PV_GBa0086N01.r | GGTATGGTGAAATTGTTGG | GAGACTGATAATTGGGTAGAGT | 325 | (TTA)4 | 54-44 |
| BMb1732s | PV_GBa0048C09.f | TGCTTCTCTCAGTATGATTC | CTTCAAGTAAACCACACAGT | 319 | (TTA)4 | 54-44 |
| BMb1733s | PV_GBa0042E13.f | CTGCAGTTACGTTAGAGTTT | ATACAGTCATATCCAGGTCTAC | 366 | (TTA)4 | 55-45 |
| BMb1734s | PV_GBa0048L21.r | AGACAGCCCAAACTCTATAA | CACGCCTGATAATGAATG | 245 | (TTA)4 | 53-43 |
| BMb1735s | PV_GBa0030M22.f | GGGGTTACAAACGAATTAC | GTAAAAGTGGAGAGGAGACTA | 273 | (TTA)4 | 54-44 |
| BMb1736s | PV_GBa0015L17.f | CAACTTCATAGCCAGTATGT | TACTAACACGCCTGTAAACT | 260 | (TTA)4 | 55-45 |
| BMb1737s | PV_GBa0002E09.r | AAGTCAACCGACAAGAAC | CGATGACTATACCCTAACAA | 130 | (TTA)4 | 54-44 |
| BMb1738s | PV_GBa0104B24.f | ACAAAACATCTCTCCCTCT | CAAGGTTGTCAGATTAACAC | 226 | (TTA)5 | 54-44 |
| BMb1739s | PV_GBa0046L23.f | CACTACACCAAACAAATTCC | TGATATCTCCATTACACACG | 348 | (TTA)5 | 54-44 |
| BMb1740s | PV_GBa0044J11.f | CTGACTTATTTTGCTGCTG | CTGGGAATGGAAAATAGG | 184 | (TTA)5 | 52-42 |
| BMb1741s | PV_GBa0040E03.f | GATGTGATGGGTGTGATA | TCTTGAGTGCTCTACACTTT | 400 | (TTA)5 | 54-44 |
| BMb1742s | PV_GBa0003O07.f | GTGTTCCTACAATGTTCTCA | CCCAGAAGATAACACTATCA | 278 | (TTA)7 | 54-44 |
