# Supplementary material for: BAC-End Microsatellites from Intra and Inter-Genic Regions of the Common Bean Genome and Their Correlation with Cytogenetic Features
Source: PLoS One. 2014 Sep 25;9(9):e101873. doi: 10.1371/journal.pone.0101873 (PMC4177843; doi:10.1371/journal.pone.0101873)
Supplement: Table S2 — Marker order per linkage group for all BMb markers both from contigged and singleton BACs. (PDF) [file pone.0101873.s002.pdf]

LG	SSR	Contig	Contig size Kb	Comments	Distance
b01h	BMb644s	0	NA	Singleton derived	0.0
	BMb1027	72	94	Contig derived	21.4
	BMb256	294	88	Contig derived	25.2
	BMb405	873	126	Contig derived	20.3
	BMb1079	349	142	Contig derived	10.9
	BMb1067	248	94	Contig derived	13.0
	BMb716s	0	NA	Singleton derived	17.6
	BMb744s	0	NA	Singleton derived	3.5
	BMb194	205	124	Contig derived	10.8
	BMb83	77	116	Contig derived	2.6
	BMb859s	0	NA	Singleton derived	2.6
	BMb1287	1198	112	Contig derived	1.9
	BMb1733s	0	NA	Singleton derived	0.0
	BMb1200	2373	79	Contig derived	8.3
	BMb513	1690	114	Contig derived	4.2
	BMb1189	2175	107	Contig derived	2.5
	BMb502	1626	103	Contig derived	4.5
	BMb290	384	96	Contig derived	0.7
	BMb213	225	78	Contig derived	20.6
	BMb670s	0	NA	Singleton derived	5.4
	BMb1162	1542	75	Contig derived	5.1
	BMb1089	382	110	Contig derived	4.3
	BMb1681s	0	NA	Singleton derived	21.0
	BMb1263	633	118	Contig derived	6.6
	BMb1675s	0	NA	Singleton derived	2.9
	BMb1672s	0	NA	Singleton derived	0.6
	BMb64	49	82	Contig derived	4.8
	BMb1631s	0	NA	Singleton derived	17.6
	BMb1230	83	124	Contig derived	3.1
	BMb1191	2255	74	Contig derived	3.8
	BMb1024	70	101	Contig derived	0.6
	BMb1125	704	101	Contig derived	5.1
	BMb1725s	0	NA	Singleton derived	2.2
	BMb1617s	0	NA	Singleton derived	3.4
	BMb1599s	0	NA	Singleton derived	13.6
	BMb650s	0	NA	Singleton derived	4.1
	BMb810s	0	NA	Singleton derived	1.9
b02d	BMb125	117	95	Contig derived	0.0
	BMb180	179	115	Contig derived	23.3
	BMb1643s	0	NA	Singleton derived	25.0
	BMb700s	0	NA	Singleton derived	16.7
	BMb1163	1543	69	Contig derived	17.0
	BMb1186	2148	99	Contig derived	12.8
	BMb768s	0	NA	Singleton derived	8.7
	BMb1137	849	80	Contig derived	7.4

	BMb80	75	118	Contig derived	13.0
	BMb1289	1239	88	Contig derived	3.3
	BMb259	311	103	Contig derived	6.7
	BMb252	282	84	Contig derived	3.6
	BMb712s	0	NA	Singleton derived	4.5
	BMb1131	725	95	Contig derived	2.2
	BMb1605s	0	NA	Singleton derived	1.9
	BMb1594s	0	NA	Singleton derived	2.8
	BMb497	1593	85	Contig derived	1.3
	BMb527	1833	96	Contig derived	4.6
	BMb1583s	0	NA	Singleton derived	0.6
	BMb97	91	124	Contig derived	0.6
	BMb420	1022	120	Contig derived	3.1
	BMb1691s	0	NA	Singleton derived	0.6
	BMb1607s	0	NA	Singleton derived	2.4
	BMb1266	713	73	Contig derived	3.0
	BMb1649s	0	NA	Singleton derived	1.4
	BMb1601s	0	NA	Singleton derived	4.2
	BMb1126	713	75	Contig derived	0.7
	BMb1192	2266	106	Contig derived	0.0
	BMb1194	2287	87	Contig derived	2.1
	BMb1286	1191	126	Contig derived	4.9
	BMb495	1579	74	Contig derived	4.1
	BMb793s	0	NA	Singleton derived	4.8
	BMb1692s	0	NA	Singleton derived	3.3
	BMb469	1428	115	Contig derived	5.4
	BMb681s	0	NA	Singleton derived	16.7
	BMb755s	0	NA	Singleton derived	40.2
	BMb802s	0	NA	Singleton derived	12.8
	BMb701s	0	NA	Singleton derived	17.0
b03c	BMb719s	0	NA	Singleton derived	0.0
	BMb581	2427	65	Contig derived	30.7
	BMb1706s	0	NA	Singleton derived	57.4
	BMb521	1782	120	Contig derived	18.3
	BMb1648s	0	NA	Singleton derived	8.3
	BMb1632s	0	NA	Singleton derived	4.4
	BMb1215	2962	71	Contig derived	3.3
	BMb339	502	102	Contig derived	5.6
	BMb1188	2167	87	Contig derived	2.9
	BMb247	272	88	Contig derived	2.0
	BMb508	1648	102	Contig derived	3.6
	BMb1203	2442	84	Contig derived	3.6
	BMb1010	26	101	Contig derived	1.4
	BMb506	1642	113	Contig derived	2.0
	BMb191	196	85	Contig derived	0.6
	BMb1113	612	100	Contig derived	0.6

	BMb2	4	93	Contig derived	1.9
	BMb1195	2298	107	Contig derived	13.8
	BMb1259	517	86	Contig derived	0.7
	BMb477	1447	145	Contig derived	0.7
	BMb1674s	0	NA	Singleton derived	5.7
	BMb1600s	0	NA	Singleton derived	3.8
	BMb1730s	0	NA	Singleton derived	0.6
	BMb590	2469	100	Contig derived	11.5
	BMb1171	1699	78	Contig derived	12.0
b04b	BMb1102	457	81	Contig derived	0.0
	BMb831s	0	NA	Singleton derived	17.6
	BMb686s	0	NA	Singleton derived	11.8
	BMb548	2031	96	Contig derived	0.7
	BMb488	1487	87	Contig derived	9.3
	BMb1101	450	70	Contig derived	6.9
	BMb1118	657	118	Contig derived	3.8
	BMb1244	213	110	Contig derived	1.2
	BMb1160	1533	71	Contig derived	3.7
	BMb66	50	139	Contig derived	5.1
	BMb866s	0	NA	Singleton derived	5.9
	BMb1719s	0	NA	Singleton derived	1.3
	BMb133	133	103	Contig derived	2.6
	BMb571	2295	74	Contig derived	1.3
	BMb353	605	127	Contig derived	0.0
	BMb1187	2154	62	Contig derived	1.3
	BMb856s	0	NA	Singleton derived	0.6
	BMb1602s	0	NA	Singleton derived	0.6
	BMb1679s	0	NA	Singleton derived	2.7
	BMb775s	0	NA	Singleton derived	10.0
	BMb43	34	94	Contig derived	31.3
	BMb857s	0	NA	Singleton derived	0.0
b05e	BMb705s	0	NA	Singleton derived	0.0
	BMb1694s	0	NA	Singleton derived	9.3
	BMb821s	0	NA	Singleton derived	5.9
	BMb698s	0	NA	Singleton derived	7.5
	BMb318	436	79	Contig derived	0.6
	BMb710s	0	NA	Singleton derived	1.3
	BMb1016	40	152	Contig derived	2.8
	BMb1182	2087	89	Contig derived	1.2
	BMb813s	0	NA	Singleton derived	3.6
	BMb121	111	125	Contig derived	6.1
	BMb1619s	0	NA	Singleton derived	10.3
	BMb611	2765	105	Contig derived	10.0
	BMb742s	0	NA	Singleton derived	13.4
	BMb250	274	86	Contig derived	1.5

	BMb560	2170	73	Contig derived	2.5
	BMb1071	267	115	Contig derived	0.6
	BMb1575s	0	NA	Singleton derived	2.5
	BMb1661s	0	NA	Singleton derived	4.4
	BMb1650s	0	NA	Singleton derived	6.2
b06g	BMb625s	0	NA	Singleton derived	0.0
	BMb1688s	0	NA	Singleton derived	10.9
	BMb519	1747	93	Contig derived	4.4
	BMb182	185	103	Contig derived	5.5
	BMb341	527	90	Contig derived	10.8
	BMb539	1891	94	Contig derived	7.6
	BMb1279	947	74	Contig derived	2.0
	BMb1108	530	128	Contig derived	0.0
	BMb1061	238	82	Contig derived	0.0
	BMb1158	1483	94	Contig derived	0.0
	BMb419	1001	90	Contig derived	6.5
	BMb1105	498	84	Contig derived	8.4
	BMb1157	1372	106	Contig derived	11.9
	BMb1682s	0	NA	Singleton derived	3.5
	BMb1705s	0	NA	Singleton derived	5.5
b07a	BMb489	1497	64	Contig derived	0.0
	BMb1148	1108	83	Contig derived	2.5
	BMb751s	0	NA	Singleton derived	19.1
	BMb1198	2339	90	Contig derived	6.1
	BMb202	212	116	Contig derived	22.3
	BMb799s	0	NA	Singleton derived	16.7
	BMb1728s	0	NA	Singleton derived	7.0
	BMb160	159	92	Contig derived	3.1
	BMb1142	957	86	Contig derived	0.0
	BMb1080	352	99	Contig derived	0.0
	BMb526	1832	110	Contig derived	0.7
	BMb621	2916	149	Contig derived	2.7
	BMb601	2606	74	Contig derived	4.8
	BMb1703s	0	NA	Singleton derived	3.9
	BMb1275	839	75	Contig derived	5.1
	BMb428	1099	87	Contig derived	15.2
	BMb1117	628	106	Contig derived	14.3
	BMb1620s	0	NA	Singleton derived	17.3
b08f	BMb1208	2569	65	Contig derived	0.0
	BMb531	1869	94	Contig derived	10.1
	BMb1309	2155	67	Contig derived	0.9
	BMb1671s	0	NA	Singleton derived	5.4
	BMb445	1142	77	Contig derived	3.9
	BMb1684s	0	NA	Singleton derived	15.4

	BMb1319	2494	76	Contig derived	4.3
	BMb1699s	0	NA	Singleton derived	6.7
	BMb386	769	104	Contig derived	9.3
	BMb1055	195	89	Contig derived	9.3
	BMb267	346	97	Contig derived	0.6
	BMb1610s	0	NA	Singleton derived	2.6
	BMb847s	0	NA	Singleton derived	3.4
	BMb174	172	99	Contig derived	0.0
	BMb277	359	74	Contig derived	0.7
	BMb1039	107	138	Contig derived	1.3
	BMb529	1845	90	Contig derived	2.5
	BMb578	2412	111	Contig derived	1.8
	BMb362	665	149	Contig derived	1.8
	BMb1736s	0	NA	Singleton derived	0.6
	BMb1668s	0	NA	Singleton derived	0.0
	BMb1229	2106	93	Contig derived	4.7
	BMb1710s	0	NA	Singleton derived	5.1
	BMb1673s	0	NA	Singleton derived	1.9
	BMb1205	2464	54	Contig derived	3.7
	BMb1297	1635	91	Contig derived	3.1
	BMb1595s	0	NA	Singleton derived	1.2
	BMb871s	0	NA	Singleton derived	6.5
	BMb475	1443	84	Contig derived	3.2
	BMb474	1443	128	Contig derived	3.1
	BMb559	2157	80	Contig derived	0.7
	BMb1614s	0	NA	Singleton derived	5.8
	BMb266	342	82	Contig derived	9.7
	BMb553	2134	93	Contig derived	10.7
	BMb1654s	0	NA	Singleton derived	26.9
	BMb1196	2307	88	Contig derived	1.9
	BMb745s	0	NA	Singleton derived	10.1
	BMb1700s	0	NA	Singleton derived	26.3
b09k	BMb773s	0	NA	Singleton derived	0.0
	BMb683s	0	NA	Singleton derived	5.7
	BMb279	367	96	Contig derived	4.2
	BMb851s	0	NA	Singleton derived	10.0
	BMb1277	888	124	Contig derived	1.2
	BMb594	2534	88	Contig derived	15.6
	BMb1645s	0	NA	Singleton derived	8.0
	BMb1685s	0	NA	Singleton derived	12.4
	BMb829s	0	NA	Singleton derived	2.7
	BMb1119	668	107	Contig derived	2.4
	BMb766s	0	NA	Singleton derived	2.5
	BMb737s	0	NA	Singleton derived	3.4
	BMb1625s	0	NA	Singleton derived	5.1
	BMb685s	0	NA	Singleton derived	4.4

	BMb598	2543	90	Contig derived	0.6
	BMb143	137	102	Contig derived	2.7
	BMb493	1544	122	Contig derived	10.6
	BMb1644s	0	NA	Singleton derived	11.5
	BMb1036	96	106	Contig derived	15.6
	BMb264	331	107	Contig derived	10.1
b10i	BMb718s	0	NA	Singleton derived	0.0
	BMb447	1154	102	Contig derived	25.1
	BMb1222	31	113	Contig derived	22.9
	BMb262	327	100	Contig derived	14.7
	BMb276	358	89	Contig derived	11.6
	BMb734s	0	NA	Singleton derived	14.8
	BMb1206	2520	95	Contig derived	7.9
	BMb532	1869	86	Contig derived	10.6
	BMb1034	92	90	Contig derived	8.4
	BMb1084	358	92	Contig derived	0.0
	BMb221	241	80	Contig derived	0.6
	BMb106	101	82	Contig derived	0.0
	BMb152	142	122	Contig derived	1.2
	BMb414	924	107	Contig derived	1.9
	BMb1051	164	79	Contig derived	5.0
	BMb96	90	139	Contig derived	19.6
	BMb302	416	103	Contig derived	5.7
	BMb1095	398	68	Contig derived	13.0
b11j	BMb730s	0	NA	Singleton derived	0.0
	BMb1722s	0	NA	Singleton derived	8.8
	BMb816s	0	NA	Singleton derived	10.9
	BMb32	27	109	Contig derived	10.0
	BMb185	187	94	Contig derived	6.7
	BMb310	426	93	Contig derived	4.7
	BMb1093	395	117	Contig derived	2.7
	BMb791s	0	NA	Singleton derived	3.1
	BMb1228	63	108	Contig derived	1.4
	BMb484	1458	117	Contig derived	0.6
	BMb654s	0	NA	Singleton derived	3.2
	BMb819s	0	NA	Singleton derived	1.9
	BMb659s	0	NA	Singleton derived	1.4
	BMb653s	0	NA	Singleton derived	3.3
	BMb588	2467	102	Contig derived	3.0
	BMb10	11	85	Contig derived	3.9
	BMb1072	291	82	Contig derived	0.6
	BMb619	2913	58	Contig derived	15.9
	BMb1074	291	82	Contig derived	0.6
	BMb1739s	0	NA	Singleton derived	2.5
	BMb1742s	0	NA	Singleton derived	3.9
